# Seven Myths on Crowding and Peripheral Vision

**DOI:** 10.1177/2041669520913052

**Published:** 2020-05-19

**Authors:** Hans Strasburger

**Affiliations:** Georg-August-Universität, Göttingen, Germany Ludwig-Maximilians-Universität, München, Germany

**Keywords:** crowding, psychophysics, perception, reading, visual acuity, peripheral vision, fovea, asymmetries, sensory systems, cortical map, vision science, visual field

## Abstract

Crowding has become a hot topic in vision research, and some fundamentals are now widely agreed upon. For the classical crowding task, one would likely agree with the following statements. (1) Bouma’s law can be stated, succinctly and unequivocally, as saying that critical distance for crowding is about half the target’s eccentricity. (2) Crowding is predominantly a peripheral phenomenon. (3) Peripheral vision extends to at most 90° eccentricity. (4) Resolution threshold (the minimal angle of resolution) increases strongly and linearly with eccentricity. Crowding increases at an even steeper rate. (5) Crowding is asymmetric as Bouma has shown. For that inner-outer asymmetry, the peripheral flanker has more effect. (6) Critical crowding distance corresponds to a constant cortical distance in primary visual areas like V1. (7) Except for Bouma’s seminal article in 1970, crowding research mostly became prominent starting in the 2000s. I propose the answer is “not really” or “not quite” to these assertions. So should we care? I think we should, before we write the textbook chapters for the next generation.

In 1962, the ophthalmologists James Stuart and Hermann Burian published a study on amblyopia where they adopted a nice and clear term when they spoke of the *crowding phenomenon*^1^,^2^ to describe why standard acuity test charts are mostly unsuitable for amblyopic subjects: On most standard charts, as ophthalmologists and optometrists knew, optotypes on a line are too closely spaced for valid assessment of acuity in all cases such that in particular amblyopic subjects (and young children) may receive too low an acuity score. The phenomenon had been reported briefly earlier by the Danish ophthalmologist Holger Ehlers^3^ (Ehlers, 1936, 1953), who was perhaps the first to use the term *crowding* in that context, and it was treated in Adler’s textbook (Adler, 1959, pp. 661–662). Because amblyopic vision—commonly known as the “lazy eye syndrome”—leads to a strangely impaired percept and is quite unlike familiar blurred vision, it has, for the purpose of illustration, often been likened to peripheral (or indirect^4^) vision, which shares that obscurity (Strasburger & Wade, 2015a). Indeed, the same phenomenon of crowding with closely spaced patterns occurs there, that is, at a few degrees of visual angle away from where one fixates. A simple example is shown in Figure 1. Viewed at arm length, the left duck is at very roughly 4° eccentricity, and, when surrounded by fellow ducks, the same duck at the right and the same eccentricity is indistinct and obscure. Note that the visibility is not a matter of the target size here, that is, it has nothing to do with acuity or resolution in the visual field. Note further that standard textbook theories based on local, bottom-up processing, invoking simple versus complex receptive field types, retinal lateral inhibition, rate of convergence/divergence of sensory neurons, and the like, will not explain the phenomenon that, as we today know, happens in the cortex (for discussions of theories see, e.g., Tyler & Likova, 2007; Pelli, 2008; Strasburger, 2014; Kwon et al., 2014; Rosenholtz, 2015; Strasburger, 2019). Simple as it is, this little demonstration—by its ubiquity in everyday natural scenes, and its simplicity (it can be shown on a napkin)—already shows that we have a very basic, general phenomenon of visual perception here, not some niche interest of vision researchers.

**Figure 1.**
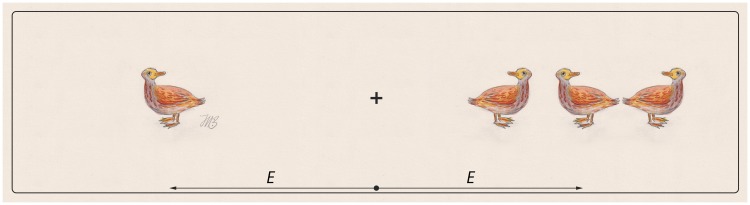
Simple Demonstration of Crowding. When fixating at the cross, the orientation for the duck on the left is seen but not that for the middle one on the right, even though the images are of the same size and at the same eccentricity. The phenomenon depends predominantly on eccentricity and pattern spacing and is mostly independent of target size. Duck painting by Ilse Maria Baumgart, Munich, 2019.

Independently, and at around the same time, the phenomenon and related phenomena were studied quite extensively in a separate research tradition, Gestalt psychology (Korte, 1923) and later in experimental psychology (e.g. Wolford, 1975; Krumhansl & Thomas, 1977; Chastain, 1982, 1983). Little did these two research communities appear to know of each other: By the time that I started being interested in crowding in 1988, there were 20 major articles on the subject, under a variety of keywords (lateral masking/inhibition/interference, interaction effects, contour interaction, surround suppression), which, more often than not, took scarce notice of those of the other line of thought (as evidenced by their references). There were only few articles at vision conferences and none in the emerging cognitive sciences or in visual neuroscience.

Things changed in the 1990s and early 2000s. Levi et al. (1985) had studied crowding in vernier acuity; Lewis O. Harvey suggested that we (myself, Ingo Rentschler, and Lew Harvey) study character crowding at low contrast and ask what mechanisms might underlie crowding (Strasburger et al., 1991; Strasburger & Rentschler, 1995). Latham and Whitaker (1996) studied the influence of four surrounding flankers on a three-bar grating, where they showed that spatial interference grew at a much faster rate with eccentricity than acuity (with *E*_2_ values^5^ only one tenth of those for acuity). He et al. (1996) pointed to the role of spatial attention, and, in particular, Denis Pelli started projects on crowding^6^ and, together with Melanie Palomares and Najib Majaj, published a seminal article, covering all the basics (Pelli et al., 2004). Crucially, however, Pelli drew attention to the fact that, contrary to common wisdom, crowding is much more important for pattern recognition than is acuity and that it overrides the latter even in the fovea,^7^ widely held to be superior *because* of its outstanding acuity in its centre (Latham & Whitaker, 1996; Pelli et al., 2007; Pelli & Tillman, 2008).

Small as it might seem, the shift of emphasis away from (inherently low-level) acuity to (inherently higher level) crowding amounts, as I see it, to nothing less than a paradigm shift. It does away with centuries of two core assumptions in visual perception (cf. Strasburger & Wade, 2015a), namely that good vision comes down to good acuity and, more generally, that a reductionist approach is necessarily and always the best way for solving a scientific problem. The *acuity myth* is everywhere. We find it in driving licence regulations (where acuity tests are often the only strict psychometric requirement for a driver’s license), or when a textbook presents a trivialized dichotomy of parvo (*P*) and magno (*M*) systems in which the *P* system is supposedly specialized on pattern recognition *because* of its high resolution and small receptive fields. Thomas Kuhn in *The Structure of Scientific Revolutions* (Kuhn, 1962) explains that research traditions in science often pervade through many decades (or perhaps centuries?), adding more and more detail to a scientific narrative until suddenly, within a few years, the viewpoint shifts radically and something new starts. The shift of emphasis in human and primate pattern recognition from acuity to crowding might just represent such a turn.

Perception is a standard, and often required, subject in psychology, medicine, and other curricula, and so there are quite a few excellent textbooks on *perception* and on the senses. A standard for covering all the senses, for example, is Goldstein’s well-known *Sensation and Perception*. Acuity, receptive fields, cortical magnification, and peripheral vision are all covered—yet it says nothing about crowding. Even more worrying, acuity and crowding are confused as shown in Figure 2. The lapse might be excused in that vision is not the author’s primary field of study. But that explanation does not transfer to the several German editions, which were edited by expert vision scientists (e.g., 7th German edition, 2008, p. 50). Another standard, *Basic Vision* by Snowden et al. (2006), a more recent, and excellent perception textbook for the visual modality, explains cortical magnification and shows Anstis’s visual demonstration of that in its first edition but also skips crowding. The same is the case in the new, 2nd edition (2012). The section on peripheral vision (pp. 117–119) shows a modified version of Anstis’s magnification chart and explains scaling and cortical magnification (the chart is the impressive but misleading version of Figure 9B discussed later here in the article, with a caption^8^ that warrants understanding why it is wrong).

**Figure 2.**
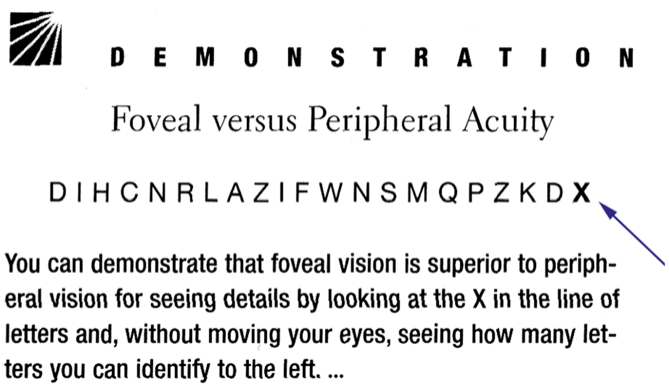
Confusion of Acuity and Crowding in Goldstein’s 6th Edition (2002, p. 57) and 9th Edition (2013, p. 43), Chapter *Neural Processing by Convergence*, Subchapter *The Cones Result in Better Detail Vision Than the Rods*. The added arrow shows where to fixate.

Mind you, the examples mentioned are already the positive exceptions. Peripheral vision and crowding are the poor relations in vision research. Out of 20 textbooks on vision that I went through published between 1970 and 2019, only 5 had some rudimentary coverage of peripheral vision (though without a term in the index), and even fewer mentioned crowding (three). The others, including the monumental, 1,800-page *Visual Neurosciences* by Chalupa and Werner (2004), the excellent and beautifully designed new *Sensation and Perception* by Yantis and Abrams (2017), and seven textbooks on computational vision, are silent on the subjects. The venerable Sekuler and Blake (1994), *Perception*, in contrast, and the brand new *Sensation & Perception* by Wolfe et al. (2019) have it right. Sekuler and Blake show and discuss Anstis’s charts on peripheral vision and crowding (see Figure 9), and Wolfe et al. (pp. 43–45) explain peripheral vision and show the well-known graph on receptor density (originally by Oesterberg, 1935), and explain crowding (p. 76) with reference to a figure from Whitney and Levi (2011).

Thus, either crowding is, after all, much less important for vision in general than those who work on that subject believe it is or now is the time that crowding will enter our textbooks and curricula. The frequent publications, talks, and symposia at vision conferences, the workshops,^9^ theses, and in short the observation that crowding is nowadays a kind of vision-research household item would suggest the latter. In that case, it matters that in the sudden flood of interest, quite a number of misconceptions on the topic appear to arise. To ensure, therefore, that these are kept at bay (or do not arise in the first place)—in particular in the perception books that are to come—here is an attempt to pinpoint a number of beliefs, or intuitive theories (Lucariello & Naff, 2019),^10^ that, upon more scrutiny, turn out to be misleading or perhaps just wrong. Note this is not about finding erroneous beliefs in the crowding literature; authors in the field rarely fall for these errors. The point is how, eventually, the key concepts for crowding will come across in, say, a textbook chapter, with its inherent need for brevity and graphicness. Assertions that seem unambiguous can turn out to be obstacles for understanding. Nota bene, the seven points are also not all of the same quality; they range from possible misunderstandings, questionable assertions, and apparent misconceptions, to clear-cut myths. Their selection reflects what I found interesting and noteworthy. Note also that, for now, the following is mostly about the isolated, “standard” crowding task—a target with singly occurring flankers. It is not about visual crowding (or crowding theories) *in general*. There will thus be further issues that might qualify as “myths,” like the hope that two mechanisms might eventually be specified that explain crowding (many authors including myself invoke two mechanisms; they are just rarely the same). I simply stopped after seven points. The article is the sixth in a series of—slightly pointed—“*myths*” presentations in vision research that I am aware of (Wade & Tatler, 2009; Rosenholtz, 2016; Bach, 2017; Strasburger, 2017a, 2017b, 2018), and I trust more will follow.^11^

Interestingly, there is no catchy German word for *crowding*, and so the English term has entered German-language scientific writing. Conversely (and on the light side), the German germane *wimmelbild* (*wimmeln* = to swarm with) is sometimes seen on English pages instead of the “Find Waldo”/“Where’s Wally” catch phrases, and in any case, those crowded images are about to develop into an art form of their own (Figure 3).

**Figure 3.**
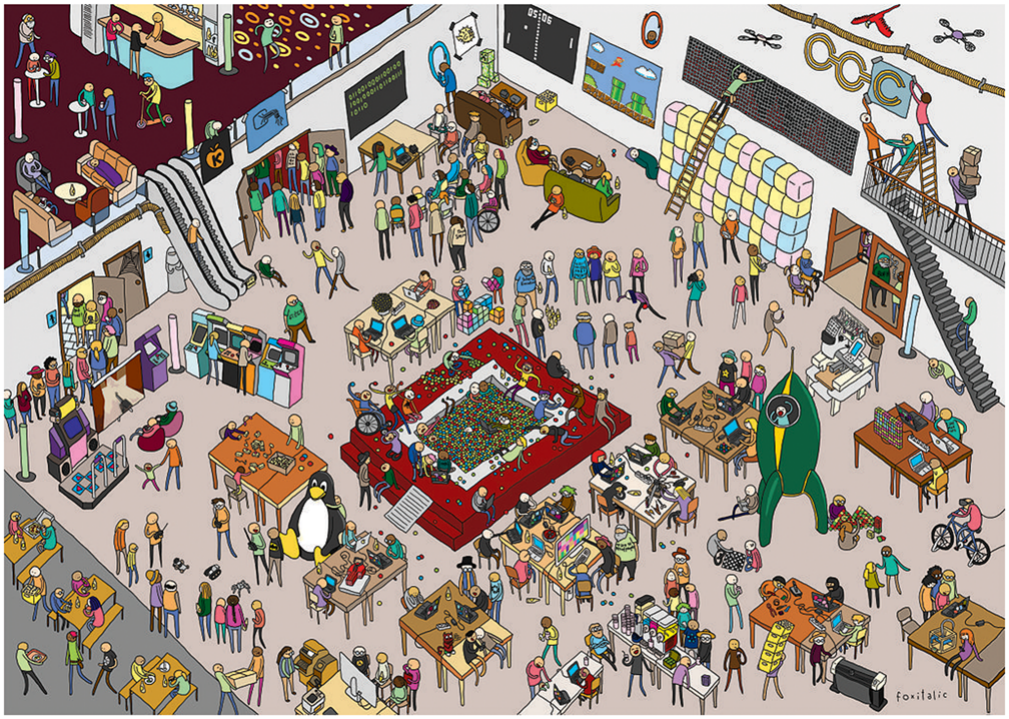
Example of a German Wimmelbild (Caro Wedekind, About the 31st Chaos Communication Congress (31C3) in Hamburg; Wedekind, 2014). Pictures like this show that visual search and crowding are connected subjects.

In medias res, one would tend to agree with the following seven statements, or wouldn’t one?

## On Bouma’s Law

*Misconception 1*: Bouma’s law can be summarized, succinctly and unequivocally, as saying that “critical distance for crowding is about half the target’s eccentricity, d≈0.5 φ (Bouma, 1970).”

In a sense that is of course correct: Bouma’s law is based on an experiment on letter triplets described in a *Nature* paper by Bouma (1970); it governs how crowding depends on the flankers’ distance to the target and specifies the minimum distance for the interference as being approximately half the eccentricity value. It operates over at least a hundredfold range. However, the simplicity of the above statement’s phrasing and the attribution are deceptive and can give rise to a number of misunderstandings. Three of these I wish to address here: (a) the law’s generality and the role of Gestalt mechanisms; (b) whether critical distance can be seen as a *critical window*, and (as the main point here), (c) what is meant by the word “*about*,” the role of a constant term, and what constitutes a law.

**Figure 4.**
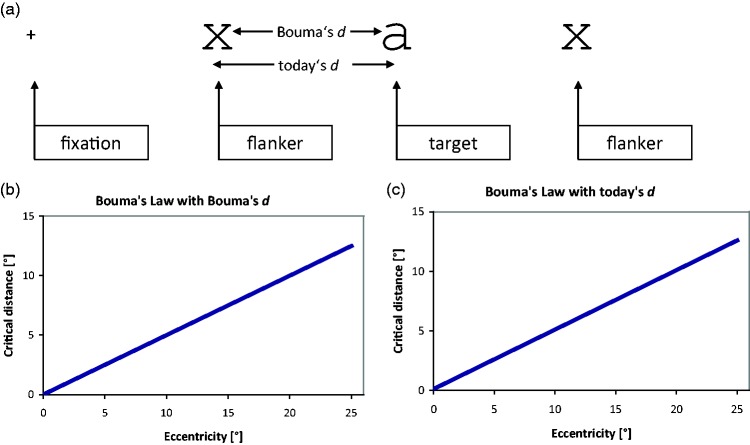
Top: Bouma’s Crowding Stimulus Arrangement. On the left is a fixation point (+), to the right of which a target letter (“a”) appears that is surrounded by two equally spaced flankers (“x”). Target and flankers are in Times Roman font, with a variable number of fixed-width spaces in between. Bottom: Bouma’s law shown over the range that crowding has been studied so far, with Bouma’s empty-space definition of critical distance (left) and today’s centre-to-centre definition (right). The difference at that scale is too small to be visible but is seen when zooming in on the article (about 10-fold; inspect the origin; or see the next figure).

1. On the first point, Bouma’s finding turned out amazingly robust and general in describing a large variety of basic crowding situations; it works with letters, low-contrast numerals, Landolt rings, gratings, and many other patterns, and amid many kinds of flankers in various numbers and orientations. It further tells us a lot about recognition of more complex patterns. After its first confirmation (Strasburger et al., 1991), Pelli et al. (2004) have studied a wide range of conditions and were the first to refer to it as *Bouma’s rul*e (p. 1143). A few years later, Pelli and Tillman (2008) discussed findings on its generality for proposing to raise Bouma’s (1970) rule of thumb^12^ to the rank of a law. Yet in spite of that impressive range of applicability, it needs to be remembered that Bouma’s law is *not* a descriptor for crowding in general. The reason for this is that human pattern recognition (see, e.g., Strasburger et al., 2011; DiCarlo et al., 2012), for which the crowding phenomenon is a central ingredient, can be subject to Gestalt mechanisms (it is worth rereading Korte, 1923, here to remind oneself of the phenomenology). Gestalt mechanisms can have the opposite effects of crowding and override the specifics of local stimulus configurations, as in the examples cited later, obeying the simple truth that the whole is generally more than the sum of its parts. So as indicated in the Introduction section, the proven and tested concept of simplifying by analytical dissection can lead astray, in particular for the case of crowding, as the isolated crowding stimulus configurations like the one in Figure 1 or Figure 4A (further below) do not predict target recognition when embedded in a larger surround. A typical Gestalt mechanism is *grouping*, by which the interference of the flankers in crowding can be eliminated or even inversed by adding a background with which those flankers group. This has been shown first by Banks et al. (1979, Figure 5) and Wolford and Chambers (1983, Figure 1) (see Herzog & Manassi, 2015, Figure 2A, and Strasburger et al., 2011, Figure 19, respectively). More recently, it has been explored systematically in Bonneh and Sagi (1999), Levi and Carney (2009), Livne and Sagi (2007, 2010), and in a series of studies by Michael Herzog and coworkers (Malania et al., 2007; Sayim et al., 2008, 2010; Saarela et al., 2009; Manassi et al., 2012, 2013; Herzog et al., 2015; see Herzog & Manassi, 2015, for review). Their message can be summarized as saying that “appearance (i.e., how stimuli look) is a good predictor for crowding” (Herzog et al., 2015, p. 1). Chakravarthi and Pelli (2011) give that view a twist in saying it is not grouping among flankers that reduces crowding but, instead, that crowding is mediated by grouping of the flankers with the target (and is unaffected by grouping of the flankers with each other).

**Figure 5.**
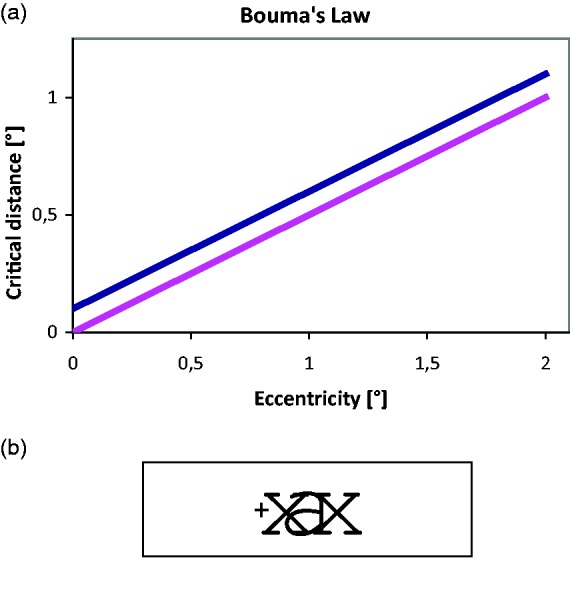
(A) Comparison of Bouma’s law with critical distance defined as empty space (pink) versus centre-to-centre (blue). (B) A degenerated stimulus configuration with overlapping flankers that would result from an incorrect statement of Bouma’s law at small eccentricity.

That said, this does not mean that, when grouping is involved, the distance between target and flankers no longer matters. All things equal, larger distance still means less crowding. The dependence on distance is changed, however, and in complicated ways that are not yet understood. Thus, grouping does not necessarily invalidate Bouma’s law; it rather challenges us clarifying how Gestalt mechanisms interact with the local situation and thereby modify Bouma’s law.

2. A second case in point concerns the influence of flankers further away than the critical distance and is related to the concept of a *crowding window*, introduced by Pelli in 2008 (Pelli, 2008; Pelli & Tillman, 2008). The proposed concept of a crowding window implies that crowding would occur *only* below the critical distance. Indeed, Pelli et al. (2004, p. 1146) suggested earlier that additional flankers on the left and right have little or no influence (they point out, however, that the data of Strasburger et al. (1991) contradict that assumption). Herzog and Manassi (2015, p. 86), in that context, phrase “Bouma (1970) showed that […] flankers interfere only when presented within a critical window […] (Bouma’s law).” That can still be read in two ways: as talking about Bouma’s original two-flanker task (for which it would be correct), (the qualifier *only* would then refer to the tested flanker distances), or as ruling out influences from outside the window (where the qualifier *only* refers to the closest vs. other flankers). However, Herzog et al. (2015, p. 1) phrase the assertion explicitly as “Crowding is determined only by nearby elements within a restricted region around the target (Bouma’s law).” That is, by the citation, the nearest-flanker-only rule is considered part of Bouma’s law. Both articles continue to show that the assertion of no influence from outside the window is incorrect and thus appears to disprove Bouma’s law (Strasburger et al., 1991, had already shown that four flankers on the horizontal meridian exert more influence than two, that is, that the assertion of no influence from outside is incorrect). Now, given that Bouma himself never talked about a multiple-flanker crowding situation and, further, that the evidence is clearly against a “nearest-only” assertion, it would seem that this assertion should not be made a constituent for a law in Bouma’s name. We thus need to pay close attention to the law’s precise phrasing and to the referenced attribution.

As to the idea of a crowding window where only the nearest neighbour counts, another interesting example for why the exact wording of Bouma’s rule (or law) matters is the article by Van der Burg et al. (2017, p. 690) on the applicability of Bouma’s rule (or law) in large, cluttered displays. The article argues that, “If visual crowding in dense displays is [not] subject to Bouma’s law, then this questions the fundamental applicability of Bouma’s law in densely cluttered displays.” (p. 693). Its conclusion is “that Bouma’s rule does not necessarily hold in densely cluttered displays [and] instead, a nearest-neighbour segmentation rule provides a better account.” (p. 690). Again, this is about *disproving*. On the surface, this might be taken as saying that Bouma’s law *as expressed in Equation 1 or 2* does not hold when displays are complex. But this is not at all what is meant in that article. What is meant (but not said in the summary) is simply that the half-eccentricity rule was not met *at the specific tested eccentricity*, and this, as a counterexample, disproves the *generality* of the rule (remember, in mathematics a single counterexample disproves a law). Only a single eccentricity was tested (because the article’s goal was elsewhere), so *linearity* or the *dependence on eccentricity* were not at stake. The results would be compatible, for example, with Bouma’s rule as stated in Pelli et al. (2004), just with a much smaller slope factor. So again, when a rule is *disproven*, it is imperative to behold the precise phrasing that is referred to (in this case the original rule).

3. As to the third of the points listed earlier, what follows here in the article is about the isolated crowding task. For that, the statement in the header sounds sensible enough and suffices as a rule of thumb, as originally intended. We can do better, however. The amazing robustness and generality across configurations of that rule suggests there is something much more fundamental about it. Starting with Pelli et al. (2007) and Pelli (2008), and in particular its discussion by Pelli and Tillman (2008), authors now frequently (and with good reason) consider it a law rather than a mere rule of thumb, equal in rank to other laws of psychophysics like Weber’s law, Riccò’s law, Bloch’s law, and so forth. Now the requirements for a law as, for example, standardly applied in classical physics are higher. One requirement is *generality*, but this is obviously a given, at least for the isolated crowding task. Another requirement, however, concerns the mathematical formulation. Not only should the mathematical description of a real-world dependency fit the empirical data, it must crucially also fulfil certain a-priori, theoretical constraints: namely to make sense for the obvious cases. That is, it must obey *boundary conditions*. As a trivial example, in the equation specifying the distance of the earth to the moon in the elliptical orbit, that distance may vary, but it must not be negative, and better not be zero. Or, for Weber’s law, zero intensity must be excluded for the principled reason that Weber’s ratio is undefined there (and the law further breaks down near the absolute threshold as explained by a statistical model by Barlow, 1957). Riccò’s law must be constrained to the area in which energy summation takes place, and so forth. A lack of such constraints is where the mathematical formulation in the header fails.

To get to that point, let us consider the qualifier *about* in the header statement. Mostly, it is understood as referring to the factor 0.5 in Bouma’s equation:

(1) d=0.5 φ

where *d* is critical distance—the minimum distance between target and flanker below which crowding occurs—and *φ* is eccentricity in degrees visual angle. Whitney and Levi (2011), in their discussion whether Bouma’s rule would qualify as a law, find the dependency of that factor on multiple influences the main issue that speaks against a law. Indeed, that factor may vary quite a bit between tasks, roughly between 0.3 and 0.7, as Pelli et al. (2004, Table 4) have listed up in their review of tasks, sometimes much more (between 0.13 and 0.7^13^ in Strasburger and Malania, 2013, Figure 9A). *Linearity*, in contrast, holds amazingly well for almost all visual tasks.^14^ So while there *is* ambiguity about the factor, that ambiguity can be easily accounted for by replacing the fixed slope factor of 0.5 in the equation by a parameter that depends on the respective task in question.

There is a more important slur, however, a limitation of the rule’s generality in range. This becomes apparent when considering the particularly important case for crowding: foveal vision and reading. The eccentricity angles (φ) in question are small there, and thus the precise meaning of a critical distance becomes important (Figure 4). Bouma (1970) specified *d* as the threshold of internal or empty space between target and flankers;^15^ today’s authors mostly prefer to specify flanker distance as measured centre-to-centre, as critical spacing then remains mostly constant across sizes as has often been shown (Tripathy & Cavanagh, 2002; Pelli et al., 2004; Pelli & Tillman, 2008; Levi & Carney, 2009; Coates & Levi, 2014; cf. also van den Berg et al., 2007, even though the independence is not perfect, e.g., Gurnsey et al., 2011).

At small eccentricities, where (by Bouma’s rule) flankers at the critical distance are close to the target, that difference of specification matters (Figure 5). With Bouma’s empty-space definition, critical distance is *proportional* to eccentricity (pink line in Figure 5A, going through the origin). With the centre-to-centre definition, in contrast, critical distance is *not* proportional to eccentricity; it is just a little bigger, by one-letter width. The difference is seen in Figure 5A, where the blue line is shifted vertically relative to the pink line. The blue line has a positive axis intercept and represents a *linear law*, not proportionality. With the centre-to-centre definition in Equation 1, the stimulus configuration would become meaningless in the fovea centre: Proportionality would imply that target and flankers are at the identical location in the centre; just off the centre, target and flankers would overlap, as shown in Figure 5B. Importantly, it is *not* what Bouma said.

To sum up the third point, in today’s terminology, Bouma described a linear law, not proportionality:

(2) d=0.5 φ+w

where *w* is letter width.^16^ We warned against this fallacy before (e.g., Strasburger et al., 2011, p. 34). Notably, Weymouth (1958) had already pointed out the importance of that difference.

Yet perhaps Equation 1 is just more elegant and appealing. Note then that Equation 2 is formally equivalent to *M* scaling (i.e., compensating for the differing cortical neural machinery across the visual field). Isn’t that beautiful? It has ramifications of its own that we wrote about elsewhere (Strasburger & Malania, 2013; Strasburger, 2019; for a review of *M* scaling, see Strasburger et al., 2011, Section 3, or Equation 9, below, and Schira et al., 2009, 2010). We will get back to that towards the end of the article, when we speak about the cortical map.

### Summary 1

In summary for Bouma’s law, if taken as a rule of thumb as intended by Bouma, the statement in the header is fine and only needs to be qualified as referring to empty space. Its attribution to Bouma (1970) is correct. It should be added in that case though that it is used as a (mere) rule of thumb. However, once we treat it as a law (as is well deserved), and in particular if it is to be disproven, more care is needed. There is probably agreement that there is something very profound to Bouma’s rule and that we are on our way to formulating a law—Bouma’s law—similar to other classical laws of psychophysics. It still needs to be sorted out, however, what its essence is. Is it the specific factor (0.5, or perhaps 0.4)? Is it the linearity, irrespective of the factor (which is my take on the matter)? Is it considered equivalent to a window? Can it be generalized beyond the isolated task, and how? Furthermore, the attributions need to be explicit because different authors put the emphasis differently. An attribution of the *law* to just Bouma (1970) without further pointers, in any case, would be incorrect and can be misleading. Importantly, the precise phrasing becomes particularly important when the rule or law is said to be disproven rather than validated.

## Crowding and Peripheral Vision

*Misconception 2*: Crowding is predominantly a peripheral phenomenon.

Crowding is of course highly important in the visual periphery. It is often even said to be *the* characteristic of peripheral vision (e.g., when amblyopic vision is likened to peripheral vision). Yet—and that is mostly overlooked—in a sense, crowding is even more important in the fovea. There, it is the bottleneck for reading and pattern recognition. Pelli and coworkers have pointed that out most explicitly (Pelli et al., 2007; Pelli & Tillman, 2008). Beware in that context that the fovea is much larger than one is mostly aware of: Its diameter is standardly stated to be around 5° visual angle (Polyak, 1941;^17^ Wandell, 1995). Note also that ophthalmologists appear to use the terms differently, referring to the 5° diameter area as the macula lutea even though the anatomical macula is again larger (diameter 6°–10°,^18^ or 17° following Polyak, 1941). Another source of confusion is the use of the term *foveal vision*. When vision scientists use that term, or speak of “the fovea,” they are typically not referring to the foveal area but are talking about the situation where the observer *fixates*; that is, they effectively refer to the foveola (having about 1.4° diameter following Polyak, 1941, or 0.5° diameter for a completely rod-free area; Tyler & Hamer, 1990). Or, indeed, they might refer to the point of highest receptor density, the very centre, sometimes called the *foveal bouquet* (Oesterberg, 1935; Polyak, 1941; Tyler & Hamer, 1990). That maximum is reached in an area of only about 8 to 16 arcmin diameter (Li et al., 2010, Figure 6^19^). The actual point of fixation (i.e., the preferred retinal locus) is furthermore not there but is between 0 and 15 arcmin away from that point (Li et al., 2010, Table 2). As a practical example, when an optometrist or ophthalmologist measures visual acuity, the result likely refers to the short moment when the gap of the Landolt ring is at the preferred retinal locus, that is, it is likely several arcmin away from the fovea’s centre. It is then that maximum acuity is achieved, and in young adults, roughly two thirds of a minute of arc are resolved at good illumination (Frisén & Frisén, 1981).

**Figure 6.**
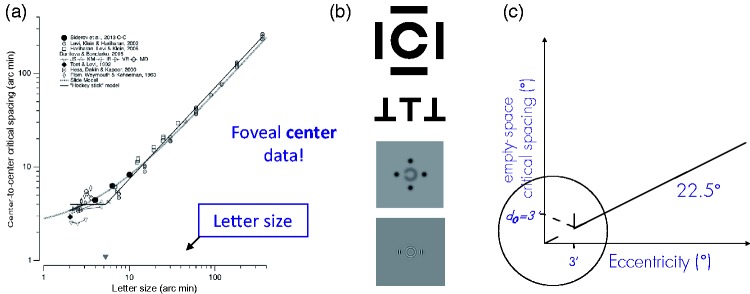
(A) Coates and Levi’s (2014) Figure 4, annotated, illustrating their “hockey stick model” that describes the dependence of centre-to-centre critical spacing on target size. The filled circles show Siderov et al.’s (2013) data for Sloan letters surrounded by bars. Note that the slope is ≈1.0, that is, an increase of letter size leads to an increase of centre-to-centre critical spacing by the same amount. The figure is annotated to emphasize that the abscissa is different from the previous figures and no eccentric data are shown. (B) A wide range of stimuli underlie the data shown in that figure, among them (top) the classical arrangement of Flom et al. (1963) or Toet and Levi's (1992) Ts (reproduced from Strasburger et al., 2011, Figure 19B, and A. Toet, personal communication, 19 Apr 2020, respectively) and also (bottom) various Gaussian and Gabor targets (Hariharan et al., 2005, from Figure 1 and Figure 2; blur intentional). (C) Possible shapes of Bouma’s law in the visual field’s very centre (with a slope of 0.5 = 22.5°) that would be compatible with the hockey stick model.

In the rest of the fovea, acuity as we all know is much lower. Phrased a bit offhand, resolving Landolt gaps is not of foremost interest for reading: Letter sizes in normal reading far exceed the acuity limit. In normal reading, letter size is somewhere around 0.4 to 2 degrees (Legge et al., 1985; Pelli et al., 2007, Figure 1)—5 to 25 times the 20/20 acuity limit.

Within the fovea, crowding is not only present off-centre (i.e., for indirect vision) but is also present in the very centre. This is what is meant by the term *foveal crowding*. Its presence has been controversial for a time but appears now well established (Flom et al., 1963; Loomis, 1978; Jacobs, 1979; Levi et al., 1985; Nazir, 1992; Polat & Sagi, 1993, 1994; Levi et al., 2002a; Ehrt & Hess, 2005; Danilova & Bondarko, 2007; Sayim et al., 2008, 2010; Lev et al., 2014; Coates & Levi, 2014; Siderovet al., 2014; Coates et al., 2018; for short reviews, see Loomis, 1978; Danilova & Bondarko, 2007; Lev et al., 2014; Coates & Levi, 2014; Coates et al., 2018). There is agreement that the interaction effect of foveal acuity targets, measured with conventional techniques, occurs “within a fixed angular zone of a few min arc” (3’–6’; Siderov et al., 2013, 2014, p. 147). However, a new study using adaptive optics (Coates et al., 2018) shows critical spacings are indeed even much smaller and only about a quarter of that range, 0.75 to 1.3 arcminutes edge-to-edge.

Whether the lateral interactions in the centre should be called “crowding” is another question. Its characteristics might (or might not) be different from those further out. Levi et al. (2002b) have it in the title—“Foveal crowding is simple contrast masking.” Coates and Levi (2014) and Siderov et al. (2014) consequently—like Flom et al. (1963)—speak of *contour interaction*. Namely, whereas crowding appears to be mostly independent of letter size (Strasburger et al., 1991; Pelli et al., 2004), that seems less so to be the case for the fovea centre, and is described by Coates and Levi (2014) as conforming with a two-mechanism model in which the critical spacing for foveal contour interaction is fixed for S < 5’ and proportional to target size for S > 5’ (Figure 6A and B). Coates and Levi (2014) call that behaviour the *hockey stick model*. Yet the new adaptive-optics data show that, for small sizes and if suitably extracted, “edge-to-edge critical spacings are exactly the same across sizes” (Coates et al., 2018, Figure 2). It thus seems that, even in the very centre, we might have standard crowding.^20^

Let us consider for a moment how the 2014 hockey stick model is related to Bouma’s law. The hockey stick model describes the situation at a single location, 0° eccentricity. For a target there of up to 5’ size, it says, centre-to-centre critical spacing is a constant 5’ (Figure 6A). The stimuli in Siderov et al. (2013) are Sloan letters surrounded by bars (having the same stroke width), so the statement could be rephrased as saying that, for Sloan letters below 5’ size presented at the very centre, the flanking bars’ midline must not be located nearer than at 5’ eccentricity to not crowd. Yet that statement appears to me as rephrasing the independence of target size in the centre, up to 5’ size.

To continue that thought, above 5’ letter size (with the target still in the centre), critical centre-centre spacing is proportional to target size according to the hockey stick model. However, because (by definition) that spacing is adjacent to the target, its centreward border will, with increasing target size, move outward at a rate of half the target size (the target extends to s/2 on each side). Thus, when *s* exceeds 5’ (where the critical gap *g* between target and flanker is smallest, at 1’),^21^ it “pushes” the flanking bar outwards. The rate at which that happens is equal to size *s*, telling from the 45° slope of the hockey stick. Gap size *g*, by the same argument, can be calculated to follow *g* = 0.3 *s* – 1’ (for s > 5’).

Taken together, the hockey stick model appears compatible with the independence of target size at 0° eccentricity (up to 5’ size) and roughly with Bouma’s law at 0° in that gap size is small (>1’) but not negative. Phrased simply, targets at 0° just need to be small enough to not come closer than 1’ to an edge at 3.5’.

The question remains whether, from the hockey stick model, we can predict what Bouma’s law would look like at very small eccentricities, that is, just off the centre. To recapitulate, at 0° eccentricity, critical gap size is about 1’–3.7’ (according to the model in Figure 6A, calculated for a target of 0.5’ up to 5’ size, with the bar at 4’) (or 0.75’–1.3’ centre-to-centre according to the new, adaptive-optics data). Now does critical target-flanker gap size, with increasing target eccentricity, increase linearly from there (as would be expected from Bouma’s law) or does it first behave differently for a few minutes of arc, and then increase (Figure 6C)? The hockey stick model, though speaking only about 0° eccentricity, appears to suggest the *latter*: By the same thought experiment as earlier, a target that is just off-centre has its boundary just a little more outward, just like that of a target at 0° that is a little larger. The nearest flanker is expected to be still at 4’ so that critical gap size might even decrease a little at first, until the target boundary comes closer than 1’, at which point standard Bouma’s law kicks in.

As a corollary, that would imply that Bouma’s law with the empty-space definition is not strictly proportionality after all but has some other behaviour below, perhaps, 4’ (Figure 6C). Note however that these derivations are tentative only, intended to illustrate how the laws might be connected. A direct test of Bouma’s law at very small eccentricities (0°–0.2°), together with how it fits in with size dependency, will be required.

### Summary 2

In summary, crowding, even though particularly pronounced in the periphery, is not just a peripheral phenomenon. It is present, and in a sense even more important, in the foveal area of around 5° diameter. The most prominent example is reading. Also, beware that saying “in foveal vision” would likely mean something else, namely the situation where the observer fixates and in which then often only the foveal bouquet counts. The term *foveal crowding*, as described for example by the hockey stick model, likewise refers to the very centre, not the foveal area.

Mind that, when we say crowding is particularly strong in the periphery, it has yet only been tested within the centre 25°-radius visual field. That is far from the “real” periphery; in perimetry and ophthalmology, the peripheral visual field refers to the area from 30° eccentricity outwards. Within that 30° radius, the area is referred to as the “central visual field.” The periphery in that sense is several times the central field in area (about seven times). It extends, on the temporal side, to around 107° eccentricity as discussed in the next section. Note in that context: Not to 90° as stated in most modern textbooks. But that is another myth story for the next section (cf. Strasburger, 2017b; Bach, 2017).

## Size of the Visual Field

*Misconception 3*: Peripheral vision extends to at most 90° eccentricity.

How far does the visual field extend to the temporal side? Crowding is particularly pronounced in peripheral vision, so we should know up to which eccentricity to look for it and thus briefly touch upon that question here.

An obvious way of finding out the size of the healthy visual field would appear consulting a standard textbook on perimetry and inspect the outermost isopter (line of equal differential luminance/contrast sensitivity) for the normal visual field. It is largest on the temporal side and extends to about 90° eccentricity. Intuitively that also seems to make sense: Light from a point in the visual field reaches the corresponding point on the retina approximately in a straight line (from the nodal points, the external and internal eccentricity angles are the same), so rays reaching the eye tangentially would not enter the eye.

**Figure 7.**
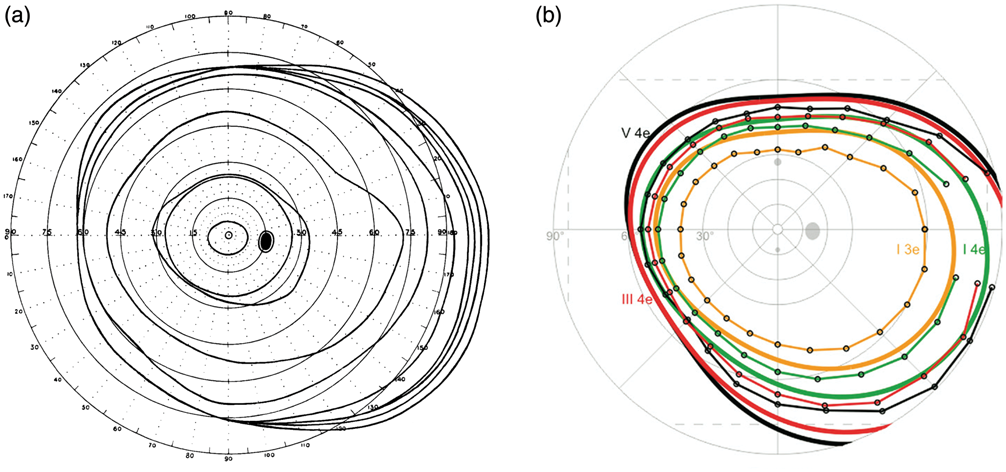
(A) The visual field, as drawn by Traquair (1938, Figure 1) in his classical book, based on the data by Rönne (1915). The outermost contour was obtained with a somewhat larger stimulus of 160 mm diameter, presented at 1 m viewing distance, that is, of 9° size. (B) A recent visual field map obtained with reaction time-corrected, semiautomated kinetic perimetry (Vonthein et al., 2007, Figure 3A).

Both assertions are, of course, wrong; the first hinges on the definition of the normal visual field; the second only works for rays entering the eye from, approximately, the front. The misunderstanding for the first assertion, that is, an interpretation of standard perimetry, is that the outermost line represents the maximum extent of the healthy visual field, when in fact it only shows the maximum extent for the *specific stimuli* used in the respective perimeter. When perimeters were developed for routine use in a clinical environment, standardization was a prime requirement. The diagnostic aim is finding impairments that warrant medical intervention, and stimuli were therefore chosen to be relatively weak to allow for sensitive testing.^22^ Furthermore, the automated cupola perimeters were, presumably to preserve space but also due to the mechanical, projection-related limitations of the stimulus excursion, designed such that the maximum angle to the side was limited to 90° eccentricity (some models had optional additional panels on the side to extend the horizontal range of measurement). However, what was forgotten over time, it seems, was that with higher contrast stimuli the visual field would extend quite a bit further out on the temporal side. The anatomical factors responsible for the visual field’s outer limits (eye brows, eye lashes, orbital bones) allow for the maximum extent in the temporal region, clearly exceeding 90°. Figure 7 shows the classic visual field diagram drawn by Harry Moss Traquair (1938) in his book on clinical perimetry, using data reported by Rönne (1915). Only just recently, there are again maps that go beyond 90° eccentricity (Figure 7B).

That the visual field extends to more than 90° on the temporal side has long been known. Purkinje (1825) found it to extend temporally up to 115°:

My measurements of the width of indirect vision indicate a temporal angle of 100 degrees (extended to 115 degrees when the pupil is enlarged by Belladonna), 80 degrees downwards, 60 degrees upwards, and the same value for the nasal angle. (Purkinje, 1825, p. 6; cited after Wade, 1998, p. 342)

Alexander Friedrich von Hueck, professor of anatomy in Dorpat/Livonia (now Tartu/Estonia; see Simonsza & Wade, 2018, for a portrait), wrote in 1840: “Outwards from the line of sight I found an extent of 110°, inwards only 70°, downwards 95°, upwards 85°. When looking into the distance we thus overlook 220° of the horizon” (Hueck, 1840, p. 84, translated by H. S.).

Hueck's is already a precise description of the visual field’s outer limits that is considered valid today. Rönne’s (1915) data were thus not surprising but provided a firm ground for Traquair’s (1938) famous map that made the visual field’s shape and size explicit (reproduced, e.g., in Duke-Elder, 1962, p. 411). For the schematic eye, Le Grand (1957, pp. 51, 52) later derives “an angle of about 109° on the temporal side.” Mütze (1961), in a standard German optometry book, shows isopters that go far beyond 90°. Similarly, Trendelenburg (1961) states as the temporal extent 90° to 100°, referring to Hermann Aubert. Schober (1970) states 90° to 110° and also points to the fact that the maximum temporal extent is not reached on the horizontal meridian but about 25° downwards (which can also be seen in Traquair’s graph; the last three references provided by B. Lingelbach, July 2017). Anderson (1987) shows a visual field that goes to 100° and has a slightly different shape (Simpson, 2017, Figure 5B). Frisén (1990), in his *Clinical Tests of Vision*, Figure 6.4 (p. 60), shows a temporal extent of 111° and explained (personal communication, 13 December 2019) that the figure represents an original observation where the outer temporal limit was obtained with a Goldmann perimeter and an eccentric fixation mark. Wade and Swanston (1991, Figure 3.4, p. 36) give as the maximum extent 104°. Wandell’s (1995) “Foundations of Vision” (which has a widely used collection of useful numbers for vision research in the inner cover) gives an overall combined angle of 200°, that is, ±100° to the temporal side. One can verify for oneself that the maximum angle is more than 90° by simply wiggling a finger on the side, from slightly behind the eye. Personally, I became aware of a possible conflict by a question from Ian Howard at VSS 2003 on my new book on peripheral vision (which I presented there and in which I claimed the extent to be 90°), when Ian Howard was about to (correctly) state 110° in his upcoming second volume of his book. Indeed, however—perhaps after our conversation—he finally (incorrectly) stated 93° (in Figure 14.1: 114°/2 + 36°) or “about 95°” in the text, citing Fischer and Wagenaar (1954, p. 370, who in turn cite Fischer, 1924 for these numbers) (Howard & Rogers, 2002, p. 2; Howard & Rogers, 2012, Vol. 2, p. 149).

Thus, by the middle of the 20th century, the maximum extent of the visual field being markedly beyond ±90° was well-established textbook knowledge. It is thus all the more surprising that this knowledge appeared suddenly lost, or perhaps considered irrelevant, at some point. The well-established German textbook on ophthalmology, Axenfeld and Pau (1992, p. 52), for example, states in its 13th edition (translated), “A normal monocular visual field extends temporally to about 90°, nasally and upwards to 60°, downwards to 70°.” Lachenmayr and Vivell’s (1992, p. 3) book on perimetry does not state the normal extent but instead shows normal maps that go to 90°. Sekuler and Blake (1994, pp. 114, 115) write, more precisely, “A normal visual field map for each eye looks like the pair numbered 1 in the accompanying figure.” The accompanying figure shows two perimetric maps that go to 90°. This is of course correct. Yet maps like these are likely misunderstood as showing the extent of the *whole* field. Indeed, Karnath and Thier’s (2006) standard German textbook on neuropsychology (p. 92) writes on the visual field (translated), “The section that we can see simultaneously without moving our head or eyes is quite large; under binocular conditions it extends to about 180° horizontally and 100° vertically.” Similarly, Diepes et al. (2007) say (translated), “1.1.2 Visual Field. The healthy visual field typically extends to about 90° temporally, 60° nasally, 50° downwards, and 40° upwards. Note these extents are, to a certain degree, dependent on the respective stimuli used” (the last sentence might hint at the field being larger with stronger stimuli). Surprisingly, many textbooks on vision do not mention the size of the visual field at all even though one would think this is basic knowledge on vision (see Table 1 for a summary; further details summarized in Bach, 2017, and Strasburger, 2017b).

**Table 1.** Books or Studies, Sorted by Publication Date, and Reported Visual Field Extent on the Temporal Horizontal Meridian.

Study	Temporal horizontal extent
Purkinje (1825)	115°
Hueck (1840)	110°
Rönne (1915)	107°
Traquair (1938)	107°
Fischer & Wagenaar (1954)	(94°)
Le Grand (1957)	109°
Mütze (1961)	(>>90°)
Trendelenburg (1961)	100°
Duke-Elder (1962)	(107°)
Aulhorn (1964)	(90°)
Schober (1970)	110°
Pöppel & Harvey (1973)	(90°)
Anderson (1987)	(100°)
Frisén (1990, Figure 6.4)	111°
Wade & Swanston (1991)	104°
Wandell (1995)	100°
Axenfeld & Pau (1992)	90°
Lachenmayr & Vivell (1992)	(90°)
Sekuler & Blake (1994)	(90°)
Karnath & Thier (2006)	90°
Howard & Rogers (2002)	93°
Diepes et al. (2007)	90°
Vonthein et al. (2007)	(∼96°)
Strasburger et al. (2011)	90°
Simpson (2017)	Review article

*Note*. Values in parentheses were not stated but are implicit in the graphs.

**Figure 8.**
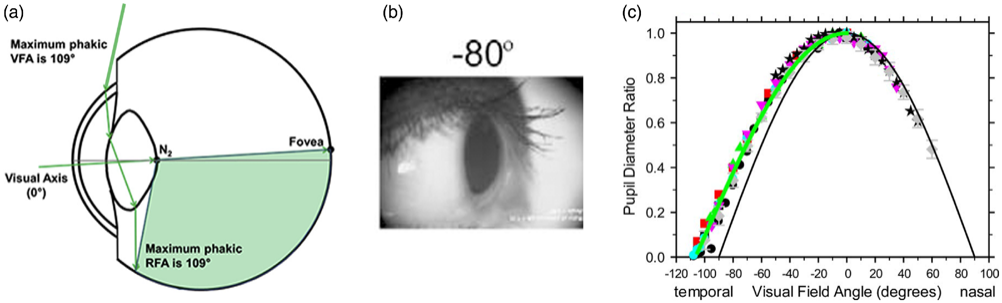
(A) Ray-trace model of how light enters the eye at the maximum angle for a 5-mm pupil (Holladay & Simpson, 2017, Figure 3A). (B) Pupil as seen from an angle of 80° on the temporal side (Mathur et al., 2013, Figure 5). (C) Aspect ratio of the pupil’s shape as seen by an observer under different horizontal angles, with data from eight different studies in the literature (coloured symbols; Mathur et al., 2013, Figure 1). VFA = visual field angle; RFA = retinal field angle.

As to the previous second erroneous assertion—the rationale that light cannot enter from the side—the answer is simply that the cornea protrudes in the eyeball so that light from the side gets refracted enough to enter the pupil. Figure 8A shows a ray-trace model by Holladay and Simpson (2017). With both a 2.5-mm and 5-mm pupil, the model predicts a maximum horizontal angle of 109° eccentricity.

To convince oneself, a nice way to visualize the effect of refraction by the cornea is looking at the eye of somebody else from the side (Figure 8B). If it were not for the refractive power of the cornea, the pupil would not be seen at all (because it is *inside* the eye), and even if it were, its circular shape would appear as a narrow vertical slit. However, when seen from the side, it appears as a vertical ellipse (Figure 8B). The maximum angle at which light can enter the eye can then be estimated from the aspect ratio of that ellipse (Figure 8C) which in that graph vanishes at around 107°.

### Summary 3

In summary, the visual field extends to about 107° to 109° eccentricity on the temporal side of the visual field, as has been known since the 19th century. The myth that it ends at 90° is likely due to technical limitations of standard perimeters for widespread clinical use and a misinterpretation of the resulting maps. It has spread to numerous textbooks since.

## Crowding and Acuity Compared

We have seen how crowding’s critical distance increases linearly with eccentricity (Bouma’s law), and how, already in the fovea, it is typically more important than acuity even at moderate eccentricities because it increases at a much faster rate (Latham & Whitaker, 1996; Pelli et al., 2007; Pelli & Tillman, 2008). How could that comparison between crowding and acuity be expressed briefly? Latham and Whitaker (1996, p. 56), who were the first to provide a direct comparison of acuity’s and crowding’s eccentricity dependence, wrote, “Spatial interference zones have a much steeper eccentricity dependency than resolution thresholds, with the extent of zones doubling in size approximately every 0.1°.” That sounds concise and convincing (but see later). For a better understanding, we should add to that an emphasis of the incorrect huge decline of acuity that we see in textbook illustrations. We further need to point out the linearity of the respective functions.

Rosenholtz (2016), who provides a recent (and very instructive) direct comparison, writes, “The slope for this crowding function is considerably higher than that for acuity, meaning that in some sense, peripheral vision degrades because of crowding faster than it does because of loss of resolution” (Rosenholtz, 2016, p. 444). This has a comparison of slopes, which implies linearity, and would just need mentioning how steep the minimal angle of resolution (MAR) function is (which is elaborated on earlier in that article). The phrase “in some sense” would also need to be made explicit for a summary. So here is a (misguided) try:

*Misconception 4*: Resolution thresholds (MARs) increase strongly and linearly with eccentricity. Crowding increases at an even steeper rate (such that crowding eventually overcomes acuity).

Before we analyse what is wrong with that summary, let us briefly consider a common fallacy about the rate of change that is seen in Latham and Whitaker’s phrasing cited earlier. It usually goes unnoticed yet has a huge effect on the steepness of critical distance’s increase (cf. Footnote 5 and 8). A “doubling in size approximately every 0.1°” implies a size for the interference zone at eccentricity *E* of 2^10^⋅*E* times the foveal size. At 1° eccentricity, that would already be 1,024 times the foveal size. At 10°, it would be 2^100^ ≈ 10^30^ times the foveal size. This is obviously not, what was meant. The mix-up is in the meaning of the *E*_2_ value (0.1° in this case), which is implicitly used here. *E*_2_ implies an *increment* by the foveal value every 0.1°, *not a ratio*. So while the foveal value is indeed doubled at *E*_2_ = 0.1°, it is not doubled again at 0.2° but is only the foveal value tripled. At 1°, it is ninefold the foveal value, and so forth. The eccentricity function would be exponential under the doubling rule, when indeed it is only linear.

Now back to the attempt of a direct comparison between the eccentricity functions for acuity and crowding (Misconception 4). For its discussion, let me decompose the statement into two assertions, one about steepness (4a) and one about the shape of the increase and whether it is linear (4b), discussed further later.

*Misconception 4a*: Intuitively, acuity decreases severely with eccentricity, and crowding increases even more steeply.

Textbooks typically characterize peripheral vision by emphasizing its decreased spatial resolution and how that is the cause for a general inferiority of peripheral vision. Goldstein’s (2002) *Sensation and Perception* explains:

Have you ever found it difficult to locate a friend’s face in a crowd? […] The reason you need to scan the crowd was that to see enough detail to recognize a face you need to focus the image of the face on your fovea […] Only all-cone foveal vision has good **visual acuity** – the ability to see details. (p. 57)

**Figure 9.**
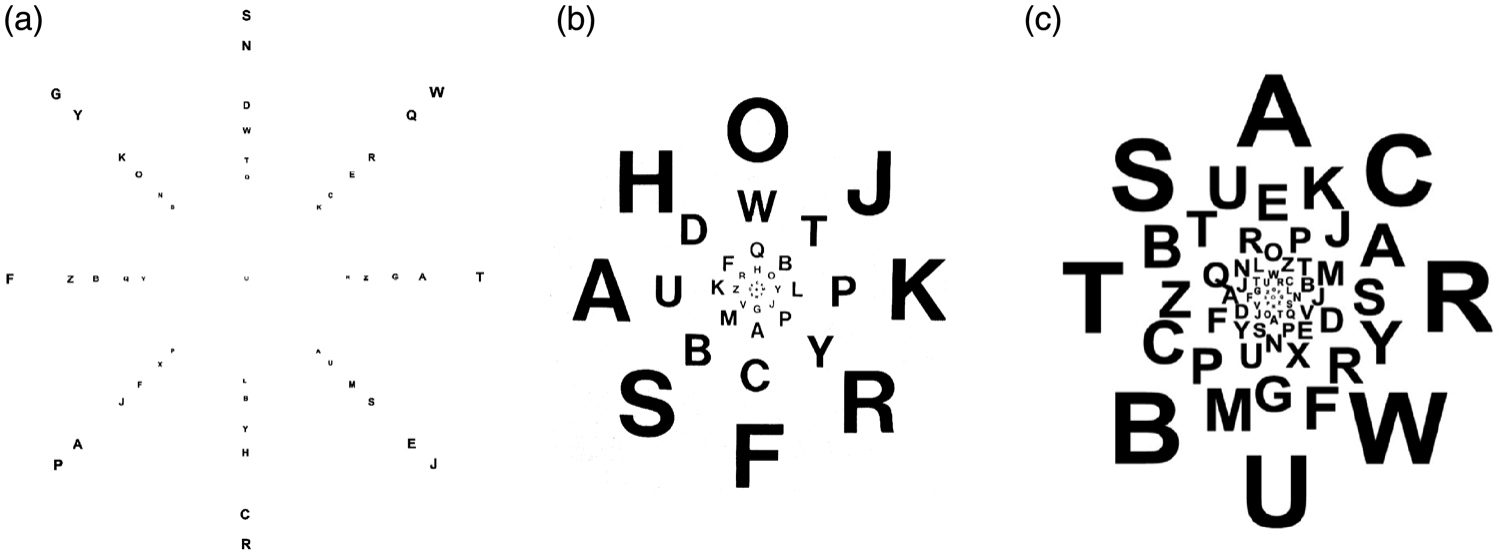
Figures 2, 3, and 4 in Anstis (1974), Illustrating Cortical Magnification. (A) Letter sizes are according to an estimate of the cortical-magnification factor (left). (B) Letters are shown at a 10-fold increased size (middle). (C) Letter sizes are the same, but more letters are added, to increase crowding (right).

**Figure 10.**
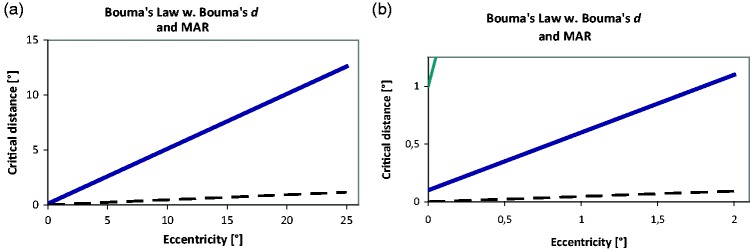
Bouma’s Law (Continuous Line, as in Figure 4A), Compared With the Increase of the MAR With Eccentricity (Dashed Line; Data From Anstis, 1974, Figure 1). Critical distance and MAR are measured in the same units (visual angle) so that these can be directly compared. The graph is shown at two scales (Part A vs. B, as in Figure 4 vs. 5 above), to illustrate that, at a large scale, the slope difference matters most, whereas at a small scale, the intercept difference is more important. Part B of the figure has an additional line starting at 1° (dark green) that shows a hypothetical *M*-scaling function scaled the same as the acuity function in the graph (see the following text). MAR = minimal angle of resolution.

Often, then, an illustration follows showing how vision is heavily blurred or degraded towards the periphery (Rosenholtz, 2016, analyses such illustrations). Now, as we all know, resolution does indeed decrease (or, conversely, the MAR increases; Weymouth, 1958). Yet, perhaps surprisingly, that happens only quite moderately. The myth of a steep MAR incline—reproduced in most every textbook that mentions the periphery—is based on the famous demonstration charts by Anstis (1974). There are three charts in that article that illustrate the change of scale across the visual field, brought about by cortical magnification (Figures 2, 3, and 4, reproduced here in Figure 9A to C). The actual enlargement of peripheral letter size to accommodate cortical magnification is shown in Anstis’s Figure 2 (Figure 9A). However, because in that chart the letters are approximately at the acuity limit and are thus hard to recognize, Anstis at the time enlarged the letters 10-fold in his Figure 3 (here Figure 9B), for better visibility. That chart looks more appealing and intuitive and, of those from Anstis’s article, is the one typically chosen elsewhere for illustrations of how the periphery differs from “ordinary,” that is, foveal, vision (e.g., Snowden et al., 2006, Figure 4.23; Rosenholtz, 2016, Figure 2; see also Strasburger et al., 2011, Figure 19G). Yet as Rosenholtz (2016) has pointed out in an enlightening article, this size enlargement at the same time dramatically overemphasizes the peripheral performance decline. This may come as a surprise but is correct. In a nutshell, it is because sizes are enlarged but eccentricities are not. We can see that from the equation given below (after discussing Figure 10). The overemphasis is by the same (whopping) factor of 10. The misunderstanding then arises because the chart is usually interpreted too literally (which Anstis probably never intended). It is a good example of how pictures can lead wildly astray.

But there is more to observe. Anstis’s second chart (here Figure 9B) is intended to show single-character recognition, illustrating the increase of the MAR. The letter spacings, measured centre-to-centre, may appear adequately spacious for preventing crowding. Yet because, by design, letter sizes are not equal, it is empty space between letters from which the influence of crowding can be estimated. An inspection of those shows that, even though for each letter the respective outward neighbour leaves around 50% of (that letter’s) eccentricity φ empty space, this is not the case for the *inward* neighbour. That neighbour only leaves between 20% and 45% of φ space. There is thus, after all, quite a bit of crowding in that graph. Consequently, the alleged effect of MAR increase in the chart is further overemphasized by inadvertent presence of crowding.

For a rough estimate of the actual rate of increase for the MAR, we can use the *E*_2_ concept and peruse Table 4 in Strasburger et al. (2011) for an overview on the empirical range of rates (see Footnote 5 and 8 for an explanation of *E*_2_). Assume for that an *E*_2_ value of 1° for Landolt acuity and a (decimal) acuity of 1.0 (“20/20”), that is, a resolvable gap size of S_0_ = 1’. These values imply a slope of 1’/1° or 1/60 = 0.017 deg/deg for the gap-size versus eccentricity function (Strasburger et al., 2011, Equation 8). Alternatively, one can inspect the data for letter acuity shown in Anstis (1974). Figure 1 in that article, or the regression equation there,^23^ shows a slope of 0.046 deg/deg for letter height. Because gap width is typically 1/5th of letter height, that translates to one-fifth of that slope (0.009 deg/deg) for the slope of MAR. In other words, we have a typical increase of roughly 1%−2% for the MAR, which is very moderate indeed.

Anstis’s third chart (Figure 9C) is an illustration of crowding. That chart is crowded, indeed! Empty spaces are obviously far smaller than the critical ½ φ. Because letter sizes are the same as before, we know acuity plays no role. Yet, again, the demo chart needs some explanation. Crowding already took place in Figure 9B, so one probably could not recognize the letters in that figure without giving up fixation. So no further effect of increased crowding will be seen. Furthermore, the large letters might lead one to believe that these sizes are what is needed in peripheral vision. One thus might wonder what, precisely, that last graph shows.

Now to the question how crowding increases with eccentricity. The increase of critical distance is certainly at a much steeper rate than it is for acuity: By Bouma’s law, critical spacing increases at a rate of ½ deg/deg, which is *30 times* the rate of increase for the MAR. It is much, much steeper. This is illustrated in Figure 10, which shows Bouma’s law from Figure 4A together with the MAR (dashed line), from Anstis’s article (1974, Figure 1).

Beware, however, that in a sense we are comparing apples to oranges here: The measure for crowding is critical distance, and target *size* does not matter much (a fivefold size change produced < 15% critical-distance change: Tripathy & Cavanagh, 2002, Figure 4; Pelli & Tillman, 2008). For the MAR, in contrast, target size not only matters—it *is itself* the measure.

There is a further caveat for our intuition in the direct comparison between crowding and MAR shown in Figure 10, related to the cortical-magnification concept: MAR is nicely described by cortical magnification (see Figure 9 in Strasburger et al., 2011), so one might assume that the same comparison as in Figure 10 holds between crowding *and cortical magnification*. That, however, is not at all the case. The reason is that cortical-magnification scaling, or *M* scaling, is a scaling concept; the reference for scaling is the foveal size threshold, that is, it is the *foveal value* that is scaled. Expressed as an equation, slope (in Figure 10) for an *M*-scaled stimulus is β=*S*_0_*/E*_2_, where *S*_0_ denotes the foveal threshold value for the task in question. The MAR line in Figure 10 is so shallow *because* the MAR’s foveal value is so small (really tiny, around 0.01°). If, however, in some experiment the foveal target is medium-sized, say 1°, the cortical-magnification-scaled results will be huge. The slope can then by far exceed the increase of crowding’s critical distance. Figure 10B includes that example; the dark green line starting at 1° and increasing steeply has the same scaling as the acuity function (dashed line) in the same graph (*E*_2_ = 0.2°).

**Figure 11.**
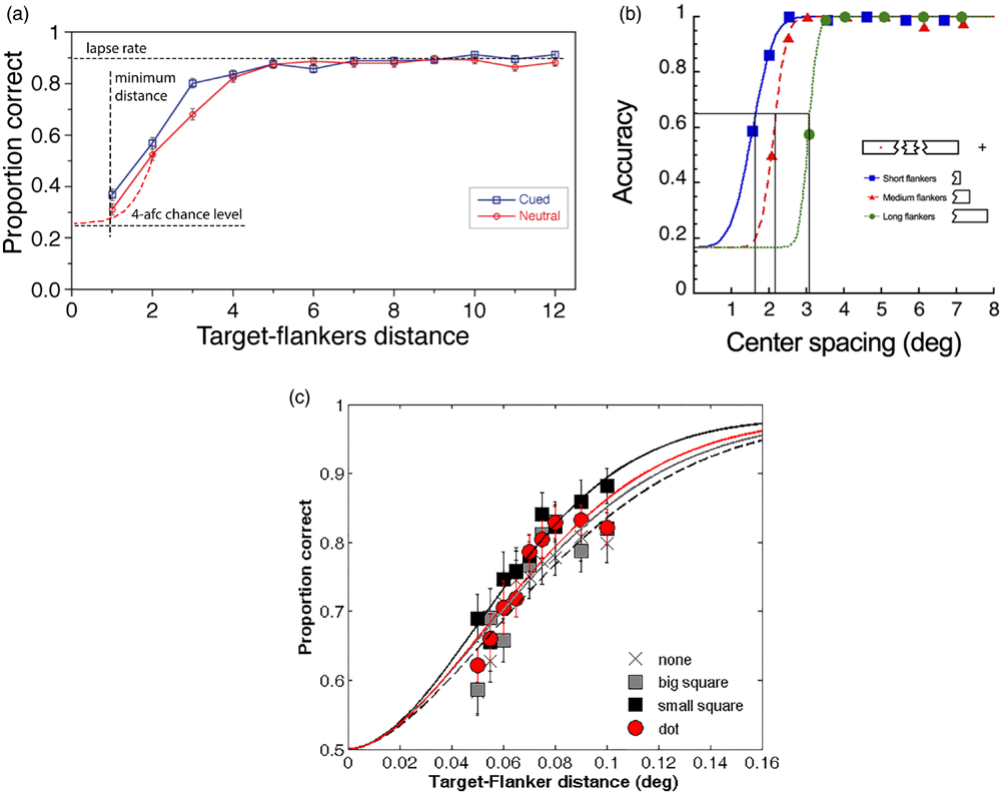
Examples of Psychometric Functions Versus Flanker Distance. (A) For letter-T recognition (the red line; disregard the blue line; eccentricity 9°; flanker distance in multiples of 0.9°). Modified from Yeshurun and Rashal (2010, Figure 5). (B) Example from Rosen et al. (2014, Figure 9A) with novel patterns that allow widening the flankers; the inset shows the stimulus and the legend; eccentricity 12°. (C) Another recent example used for quantifying spatial attention (Albonico et al., 2018, Figure 4). The four conditions refer to the kinds of attentional cue used in the study; only “*none*,” that is, the no-cue condition, is relevant here. Foveal view.

### Summary 4a

In summary for the function’s steepness (Misconception 4a), the decrease of spatial resolution towards the visual periphery is rather modest and is generally overrated in its implications. Crowding’s critical distance, in comparison, does not just increase a little “more steeply”—the difference is huge. Crowding is thus generally much more important as a limit to pattern recognition, even already in the foveal area. Visualizations of decreased acuity in the visual periphery in textbooks or in the grey literature are often misleading, as are visualizations of crowding.

#### Psychometric Function Versus Flanker Distance

Now back to the meaning of “in some sense, peripheral vision degrades because of crowding faster than it does because of loss of resolution” (Rosenholtz, 2016, p. 444). When we compare crowding with acuity, we do this by referring to their respective spatial characteristics. For crowding, this is its critical distance, and for acuity, it is acuity’s inverse, MAR. Both increase linearly with eccentricity and can be compared by their respective slope (as shown in Figure 10). Yet when we think about crowding’s *effect* on perception, like on word recognition, critical distance is somewhat of a technical aside, and we would like to say something like:

*Tentative Statement 4b*: Crowding, in its extent, increases steeply (and linearly?) with eccentricity.

That statement is still ambiguous with respect to the meaning of *extent*, and there is something fundamental about that ambiguity. “Extent” refers to two rather different domains, *intensity* (magnitude), or *space* as already elaborated on by Fechner in his classical distinction of *intensive* and *extensive* sensations (Fechner, 1860, Chapter IV, p. 15). By its standard definition and if we ask about the perceptual effect, the extent of crowding is understood as the *reduction of recognition performance* brought about by the presence of flankers. It is thus measured along a dimension that is different from the spatial dimension shown in Figure 10. For quantifying that extent, we need to convert *critical distance* to a measure of *recognition performance*.

To do that, we require the psychometric function for letter recognition versus flanker distance. A suitable performance measure is *percent correct (p_c_)*. Another well-suited performance measure would be the *contrast threshold* or *threshold elevation*, which has greater dynamic range and avoids floor effects (Strasburger et al., 1991; Strasburger, 2001a, 2001b; Pelli et al., 2004; Strasburger, 2005; van den Berg et al., 2007, cf. Figure 8 there; Strasburger & Malania, 2013). For the present purpose, however, we will stick with *p_c_*.

It is surprisingly difficult to find data for that in the crowding literature, even though it is basic for letter crowding. For the present purpose, we can look at data from Yeshurun and Rashal (2010, shown in Figure 11A, red line) that were collected as a baseline for a different research question. The task was recognizing the orientation of a grey letter “T” on a darker background amid flanking letters “H” below and above, at variable flanker distance (size: 1.05°×1.05°, Michelson contrast: 10%; eccentricity: 9°). There were four possible orientations, so chance level was 25%. The figure is modified for didactic purposes, with both axes starting at zero and dashed lines added to indicate chance level and minimum flanker distance. The red dashed line further shows the likely shape of the psychometric function at low flanker distances (because proportion correct *p_c_* cannot go below 25% as would be implied by the connecting straight lines). Figure 11B and C shows two further examples for the psychometric function versus flanker distance from other labs (Rosen et al., 2014, Figure 9A; Albonico et al., 2018, Figure 4).

**Figure 12.**
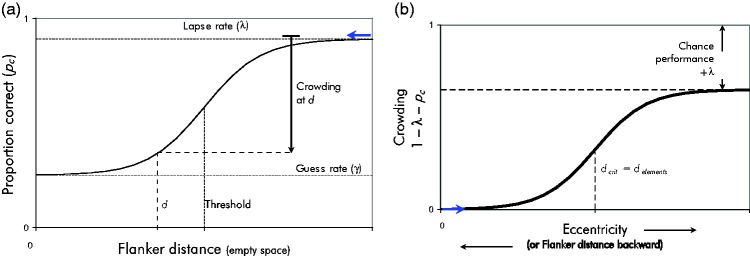
Schematic Depiction of Crowding as Defined Standardly, That Is, as the Impairment of Recognition Performance by the Presence of Flankers. (A) Psychometric function for proportion-correct performance in a crowding task, as in Figure 11. The effect of crowding, at some flanker distance *d*, is seen as the downward arrow on the right, starting from best performance (1–λ). (B) Crowding as in Part A, but now as a function of eccentricity. Part B results from Part A by mirroring the psychometric-function graph both horizontally and vertically and rescaling the *y* axis appropriately. The blue arrow serves as a graphical aid.

From that psychometric function (*p_c_* vs. flanker distance), together with Bouma’s law (which describes critical distance vs. eccentricity), we can then infer how, in principle, crowding behaves with increasing eccentricity. Note first that, for a general, principled answer to that question, distances between objects can be assumed as being, on average, independent of visual eccentricity. Examples where that is approximately the case would be letters on a printed page, or people in a crowd. Assume further that in the viewing direction that distance is above the critical crowding distance so that recognition is unaffected by crowding. Performance *p_c_* is then at its best, namely at 100% minus the lapse rate λ (top right in Figure 11). Figure 12A shows the same function schematically, to explain terms. It shows proportion correct (*p_c_*) versus flanker distance with the empty-space definition. Performance that would be obtained without flankers is the same as that obtained at sufficiently large flanker distances, that is, is 1*–*λ. Crowding, as standardly defined as the reduction of that performance by the presence of flankers, is shown as the downward arrow from that level. That reduction, that is, the length of that arrow, is 1 *–* λ *– p_c_*.

Now, to answer the question how crowding changes with eccentricity, the reduction is shown (in the upward direction) in Figure 12B. The figure is obtained from Figure 12A by rescaling the *y* axis and mirroring the graph both horizontally and vertically so that crowding (the downward arrow in Figure 12A) now goes upwards, and flanker distance *d* goes backwards. The *y* axis shows crowding, as standardly defined.

Finally, observe that Figure 12B can be reinterpreted as showing eccentricity *φ* or critical spacing *d_c_* instead of *–d* on the x axis: The psychometric function in Figure 11 or Figure 12B shows proportion correct versus (*d–d_c_*), that is, versus flanker distance minus critical distance:

(3) $$p_{c}=\Phi \;\left(d-d_{c}\right)$$

where Φ is a sigmoid function. Crowding is then

(4) $$c=\text{1}-l-p_{c}=\text{1}-\lambda -\Phi \left(d-d_{c}\right)$$

Because the distance *d* between objects is assumed to be a constant and critical distance *d_c_* is variable (it varies with eccentricity), this is a function of *–d_c_* (i.e., of *d_c_* going backwards), centred at the mean object distance *d* (as in Figure 12B). Critical distance, expressed as empty space, is proportional to eccentricity *φ* by Bouma’s law (Equation 1):

(5) $$d_{c}=\beta \;\;\varphi$$

with a scaling factor *β* around 0.5. The resulting function for crowding versus eccentricity is thus

(6) c=1−λ−Φ(d−β φ)

as shown in Figure 12B.

For an intuitive understanding, inspect Figure 12B again, starting from the left (as indicated by the little arrow). In the fovea centre, there is no crowding (*c* = 0) for the average task (like reading this article). When eccentricity is increased, critical distance (understood as empty space) increases proportionally, whereas recognition performance stays unaffected because critical distance is below the objects’ distance. However, at some eccentricity (shown as a vertical dashed line), critical distance first becomes equal and then larger than the distance between the objects in the scene. Crowding increases rapidly there, according to a sigmoid psychometric function like that in Figure 11 or 12A. A little further out in the visual field, behaviour is limited by chance performance and does not change further.

Crowding, understood in the standard sense as an effect, thus increases by a sigmoid, psychometric function with eccentricity for any given flanker distance. The same logic can be applied to acuity or the MAR (as reduction of visibility), but this is left to the reader.

### Summary 4b

In summary, crowding’s *spatial* extent (critical distance) increases linearly with eccentricity. Yet crowding’s extent, or magnitude, understood in the standard way varies by a sigmoid function: Up to some small eccentricity, in most scenes, there is no crowding at all (because adjacent contours are sufficiently far away). A little further out, there is suddenly full crowding (Figure 12B). Crowding—when understood in the standard way—cannot be compared with the MAR or acuity because, even though both are behavioural measures, they are measured on different physical dimensions (proportion correct vs. stimulus size or its inverse). It *can* be compared, however, with the *effect* of the MAR or acuity, for example, on visibility, and that is meant when we say, one overrides the other.

## Crowding Asymmetries

**Figure 13.**
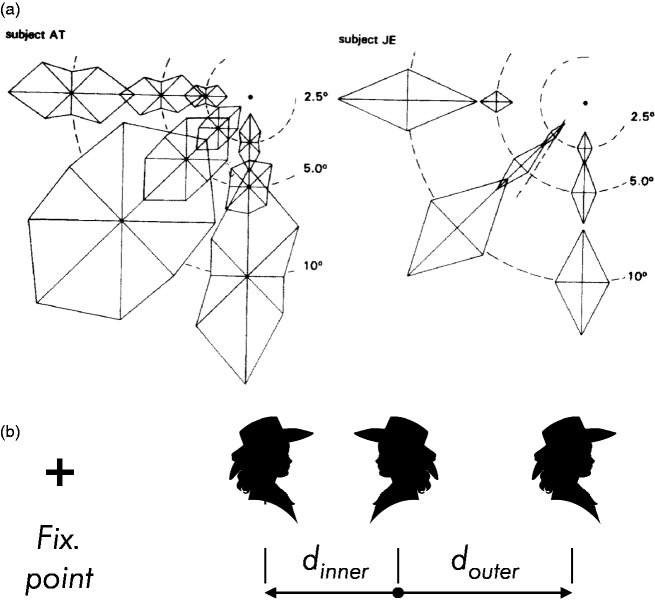
Crowding Asymmetries. (A) Radially elongated interaction fields for two subjects from Toet and Levi (1992, Figure 6), showing the well-known radial-tangential anisotropy where flankers on a radius from the visual field centre exert more influence than those arranged tangentially. (B) The inner-outer asymmetry, first studied by Mackworth (1965), refers to a different critical distance of the more peripheral versus the more central flanker. It will lead to asymmetrically elongated interaction fields.

The influence of flankers in crowding depends on where in the visual field the flankers are relative to the target, and where the target is. The effects of that are known as *crowding asymmetries*. The one best known is the radial-tangential anisotropy described by Toet and Levi (1992), where flankers on the radius from the visual field centre to the target exert more influence than those arranged tangentially, leading to the well-known, radially elongated interaction fields (Figure 13A). This asymmetry is highly reliable and has been replicated many times (Petrov & Meleshkevich, 2011a; Kwon et al., 2014; Greenwood et al., 2017), including its counterpart in the cortical map obtained with functional magnetic resonance imaging measures (Kwon et al., 2014). Another robust asymmetry in crowding refers to the location of the target, for which it has been shown that crowding is stronger in the upper than in the lower visual field (He et al.,1996; Petrov & Meleshkevich, 2011a; Fortenbaugh et al., 2015; Greenwood et al., 2017).

In the present context, however, I wish to draw attention to an asymmetry where it turns out that it is much less clear-cut than the ones mentioned earlier: The inner-outer (or “in-out”) asymmetry, which compares the influence of a flanker closer with the visual field centre to one more peripheral.^24^

*Misconception 5*: Crowding is asymmetric with respect to the effects of the inward versus the outward flanker, as Bouma (1970) has shown, the more peripheral flanker being more effective (inner-outer anisotropy).

Admittedly, as with some of the previous statements, authors in the scientific literature would not state that summary in this way.^25^ Researchers familiar with that anisotropy will further not believe that that is all to be said. However, when it comes to extracting a simplified account of that point, say for a textbook or other teaching material, or even for researchers new to the field, there is a danger that this could be the general impression that pervades.

**Figure 14.**
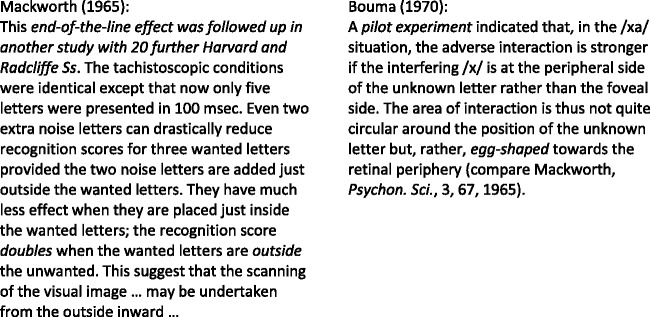
Quotes on the Central-Peripheral (Inward-Outward, “In-Out”) Asymmetry of Crowding, by Mackworth (1965) and Bouma (1970). Emphasis added.

Let us first address who is credited for that asymmetry. It often appears that the finding is credited to Herman Bouma, be it his famous *Nature* letter from 1970 or the more extensive article from 1973 (Bouma, 1973) which is both incorrect. Indeed, Bouma (1970) does mention the asymmetry, but he also warns that those were only pilot data on the asymmetry, and he notes it only as an aside at the end of the letter. The credit must go to Norman Mackworth (1965) instead: Mackworth reported the asymmetry several years earlier, and it is he to whom Bouma refers, both in his 1970 and his 1973 article (Figure 14).

**Figure 15.**
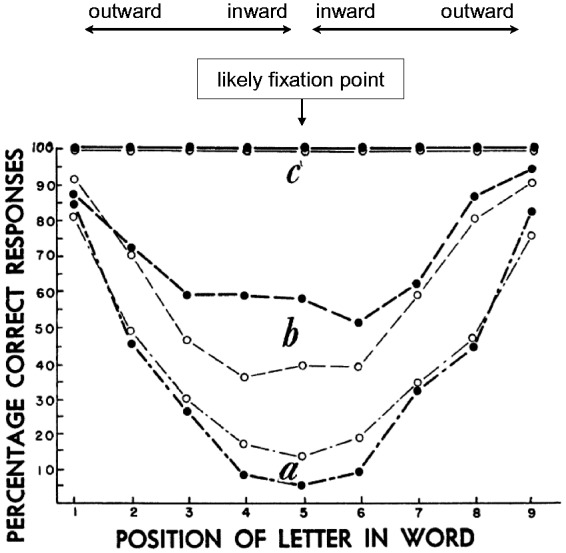
The End-of-the-Word Effect to Which Mackworth (1965) Refers (Haslerud & Clark, 1957, Figure 1). Letter recognition in 7.6°-wide nine-letter words. Open symbols: women; filled: men. *a*: fragmentary responses; *b*: incorrect; and *c*: correct. Note that both the last and the first letter are *outside* in the visual field.

Mackworth’s observation was derived from what he calls an end-of-the-line effect (referred to in the quotation), related to an end-of-the-word effect as shown, for example, by Haslerud and Clark (1957)^26^ to whom he refers in the article. Because inward/outward as referring to a word versus to the visual field are often confused (and interact with one another), the difference is illustrated in Figure 15 (Haslerud & Clark, 1957, Figure 1). Performance for the recognition of individual letters in a word depends heavily on its respective position *within the word*. Even though subjects in Haslerud and Clark’s study fixated on the words (probably somewhere near their centre; Rayner, 1979), recognition for the first and last letter (i.e., those located most peripherally) was best, followed successively by the more inward ones. Word length was about 7.6° visual angle, so letter width was around 0.6°, and the location of the first and last letter was at about ±3.5° eccentricity. Thus, already in these early experiments, the influence of eccentricity (i.e., reduced acuity) was clearly outweighed by less crowding for the first and last letter due to the adjacent empty space (Shaw, 1969; Estes & Wolford, 1971). Bouma (1973) reported a similar result, which is discussed by Levi (2008). Precursors of Haslerud and Clark (1957) for such experiments were by Benno Erdmann and Raymond Dodge (Erdmann & Dodge, 1898), and Julius Wagner (Wagner, 1918; e.g., on p. 53, he describes the better visibility of the first and last letter); see Haslerud and Clark (1957) and Korte (1923).

Bouma has also not really followed up much on the inward-outward asymmetry in the visual field; it is the left-right asymmetry and the recognition of inward versus outward letters *in a word* that he writes about in 1973 (Bouma, 1973; see Figure 14 for the difference). The inward-outward asymmetry has instead been thoroughly investigated by Estes and Wolford (1971), Estes et al. (1976), Krumhansl (1977), Banks et al. (1977), Chastain and Lawson (1979), and Chastain (1982, 1983) (and more recently by Bex et al., 2003, Petrov & Popple, 2007, Petrov et al., 2007, Dakin et al., 2010, Farzin et al., 2009, Dayan & Solomon, 2010, Petrov & Meleshkevich, 2011b, and others). Unfairly, the older articles often get no credit in the vast current crowding literature (for reviews of the asymmetries, see Strasburger & Malania, 2013, Strasburger, 2014, Levi, 2008, and Dayan & Solomon, 2010).

So, in summary for that point, crowding is asymmetric with respect to the influence of the more peripheral versus the more central flanker. That has been shown first by Mackworth (1965) in the context of an end-of-the-line effect and has been followed up by authors from experimental psychology like Estes, Krumhansl, and Chastain in the 70s and 80s, and later in vision research.

### Direction of the Asymmetry

Let us now get to the asymmetry itself and whether “crowding is directed to the fovea” (Petrov & Popple, 2007). There appears to be wide agreement that in the central-peripheral asymmetry (inward/outward in the visual field), the more peripheral flanker exerts more “adverse interaction” than the more central one (as Bouma, 1970, has put it). Bouma thus suggests that “the area of interaction is […] egg-shaped towards the retinal periphery” (p. 178), and this fits together well with the radially elongated interaction zones drawn by Toet and Levi (1992).^27^

But that unanimity is deceiving—the conclusion that the more peripheral flanker is always the more effective one is not that clear-cut as regularly suggested. Even though the superior recognizability of the peripheral flanker and its greater adverse effect on target recognition are probably uncontroversial, the consequences of that for crowding are unclear. The opposite asymmetry was reported by Chastain (1982), who found that with increasing similarity of target and flankers, the *inward* flanker leads to more impairment of accuracy, that is, in that respect plays the more important role. He further pointed out that the confusability increases with eccentricity. Furthermore, when Chastain (1982, p. 576) reanalysed Krumhansl’s (1977) data, they also supported the reverse asymmetry, counter to what was stated in her publication.

**Figure16.**
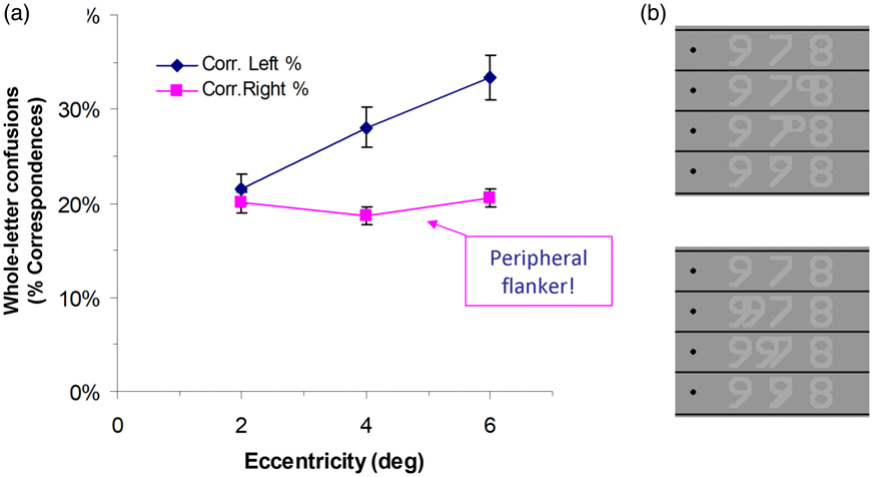
(A) Reverse Asymmetry in a Crowding Task Reported in Strasburger and Malania (2013, Figure 8A; modified). Confusions with the more central, but not the more peripheral, flanker depend on eccentricity. (B) Cartoon, as a memory aid for the mechanisms: (top) a peripheral letter part moving inward; (bottom) the more central flanker moving outward. Note that the cartoon does not quite capture the effect of features because these are a much more general concept than pattern parts.

An opposite asymmetry was further reported more recently by Strasburger and Malania (2013), with an informal model for explanation in Strasburger (2014). The data there (shown here in Figure 16A) are from a reanalysis of results for the character-crowding task in Strasburger (2005). Part of the crowding effect (up to 30%) was shown to result from whole-character confusions between target and a flanker. Contrary to our expectations, it turned out that confusions with the *inward* flanker were more frequent than with the outward one. Moreover, that difference depended on eccentricity; it increased with eccentricity for the inward, but not the outward, flanker. Note that, because whole-letter confusions are not the only reason for crowding, such a result does not contradict a stronger net inhibitory effect of the more peripheral flanker under suitable conditions.

Several formal and informal theories have been put forward to explain the central-peripheral asymmetry in crowding. Estes et al. (1976, p. 1), for example, distinguish item errors and “errors reflecting loss of positional information” and, with respect to the latter, conclude that “transposition errors exhibit a pronounced peripheral-to-central drift.” Chastain (1983, p. 154) suggests, “features from the peripheral nontarget could be mislocalized in a foveal direction to the target position.” Motter and Simoni (2007) and Nandy and Tjan (2012) invoke the laterally smaller representation of critical distance on the cortical map, though that account was shown to be insufficient as an explanation by Petrov and coworkers (Petrov et al., 2007; Petrov & Meleshkevich, 2011b). Petrov and Meleshkevich (2011b) present evidence that the inner-outer asymmetry might be due to an inherent inner-outer asymmetry of (sustained) spatial attention: (a) The outward asymmetry mostly disappeared in diffused relative to focused attention, and (b) manipulation of the spatial-attentional conditions showed that the attentional field itself (the “spotlight”) was shifted outward in the visual field. Note that spatial attention in Petrov and Meleshkevich’s study, by its implementation, refers to *sustained* spatial attention, as in Strasburger and Rentschler, 1995, He et al., 1996, Strasburger, 2005, not to *transient* spatial attention as in Strasburger, 2005, or Strasburger and Malania, 2013 (for the distinction, see Nakayama & MacKeben, 1989).

However, none of these models attempts to explain the conflicting evidence with respect to the inward-outward asymmetry. An explanation is needed how whole-letter confusions can have *opposite* properties than feature misallocations. The additional suggestion in Strasburger (2014) is to account for those conflicting asymmetry results by adding the influence of a mechanism not yet much considered in the crowding literature: feature binding as a part of the neural network dynamics in pattern processing (von der Malsburg, 1995). This computational concept is not necessarily linked to attention (i.e., it is not to be understood in the sense of Treisman & Gelade, 1980) and is not quite captured by Treisman’s (1996) “part binding” category. Features in that framework could be as in Wolford’s (1975) feature-perturbation model, which in turn were taken from Lindsay and Norman (1972; there were seven types of features there, including vertical lines, acute angles, and continuous curves). Features to be considered should be of the same colour because crowding characteristics change when flankers have different colour or contrast polarity (Pelli et al., 2004). Greenwood et al. (2012) discuss models of how binding could be related to crowding, and Yu et al. (2012) present a more recent discussion what the suitable candidates for features in word recognition could be.

Now, according to hitherto proposed accounts for explaining crowding, like Wolford’s (1975) classical feature-perturbation model, or modern statistically constrained pooling theories (Balas et al., 2009; Dakin et al., 2010; Freeman et al., 2012; Keshvari & Rosenholtz, 2016), flanker attributes get mixed in with the target letter in the crowding task, such leading to “false” percepts. Such models do not (and perhaps should not) distinguish between (erroneously attributed) individual features and (confusions with) whole characters. Indeed, Dakin et al. (2010), for example, show that whole-letter confusions can arise from interactions between features.^28^ Yet there is quite a bit of evidence that whole-letter confusions are perhaps often not just the sum of feature misallocations (Estes et al., 1976; Wolford & Shum, 1980; Strasburger et al., 1991; Huckauf & Heller, 2002; Chung et al., 2003; Strasburger, 2005; Vul et al., 2009; Strasburger & Malania, 2013). Observe that, for explaining the conflicting evidence with respect to the inner-outer asymmetry, we need *different* treatment of whole characters versus features. This is where I suggest the concept of binding comes in and further suggest that it is location dependent. Binding, whichever way implemented, is an algorithm, or system characteristic, that decides which features belong together and which do not. The proposal is now that such feature binding *decreases with visual eccentricity*. Inward flankers would thereby be more “stable” and would tend to interfere as a whole. Peripheral flankers, in contrast, would tend to mix-in features with the target (Figure 16B).

This is not to say that confusions, in whole or in part, are the whole story. Crowding mechanisms other than confusions do play a part and might further be stronger more peripherally, compared with more centrally. They could lead to a stronger overall interference of the peripheral flanker, consistent with the majority of findings on the asymmetry.^29^

### Summary 5

Crowding is not isotropic; the effects of flankers depend on their location relative to the target. The best known anisotropy is the radial-tangential kind, described by Toet and Levi (1992), where flankers along a radius from the visual field centre to the target have less effect than those tangential to that radius such that interaction fields are elongated along that radius. Yet there is another rather powerful anisotropy, the inner-outer or central-peripheral kind, where the more peripheral flanker has overall more adverse effect on recognition than the more central one, which leads to the (elongated) interaction fields being *asymmetric* along the radius. It was first described by Mackworth (1965), with many more articles following up to today. Bouma (1970) played little role here (they were only pilot data), as did Bouma (1973; because of its different meaning of inward/outward).

However, what is mostly overlooked in this context is that the direction of the asymmetry depends on the kind of effects in question. For the kind of report errors that depend on the similarity with a flanker, the asymmetry can be reversed, now with the more central flanker being the more important. This has been shown first by Chastain (1982) and in Strasburger and Malania (2013) and can be seen in the data of Krumhansl (1977). Models of crowding do not yet cover that reversed asymmetry, but a possible route has been proposed by Strasburger (2014).

## Crowding in the Cortical Map

*Misconception 6*: Critical crowding distance corresponds to a constant cortical distance in V1 and other primary visual cortical areas.

We now go from visual psychophysics to cortical neurophysiology. Crowding is a cortical phenomenon; this is known since Flom et al.’s (1963) dichoptic experiments. We further know (since Inouye, 1909) that the primary visual cortex is retinotopically organized, that is, neighbouring points in the visual field project to neighbouring points in the primary visual cortex (and in later areas up to V4). We thus speak of the *cortical map* (see Schira et al., 2010, or Schira et al., 2012, for intuitive graphics). Now, crowding is about neighbourhood in the visual field and how close visual objects are. The question that then arises naturally is how close are these objects’ representations in the cortical map? In particular, what are the critical distances for crowding in the cortical map(s)? Or, what is the equivalent of Bouma’s law in the primary visual cortex?

**Figure 17.**
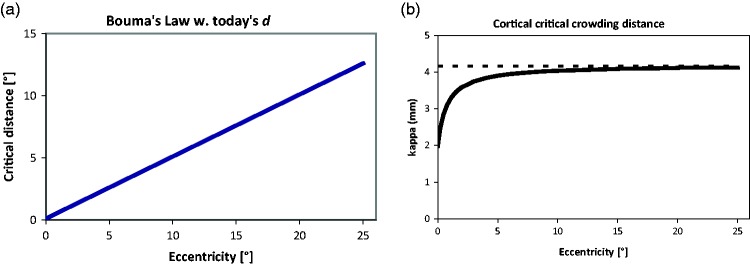
(A) Bouma’s law in the visual field and (B) its cortical equivalent, that is, how it translates to the cortical map in a primary visual area (Strasburger, 2019, Figure 8; see there for the specific parameters chosen for the estimation and note that kappa depends upon these).

Levi et al. (1985, Figure 13) found critical distance for a vernier target to be largely a constant in the cortex (∼1 mm) by applying *M* scaling with the *E*_2_ concept (they use a transformed eccentricity, E* = E + E_2_, with *E*_2_ = 0.8° for cortical processing and *E*_2_ = 2.5° for retinal processing). Motter and Simoni (2007) more generally proposed that Bouma’s law translates to a *constant* critical distance on the cortical map above 10° eccentricity, that is, the linear increase in the visual field translates to a constant in the cortex (see the dashed line in Figure 17B). Interestingly, however, their Figure 7 shows a nonconstant curve, similar to the one derived in Strasburger (2019) shown later (Figure 17B, continuous line). Pelli (2008) presented a mathematical derivation of that constancy, based on Schwartz’s (1980) logarithmic cortical mapping rule (note that it is Fischer, 1973, who should really be cited for the log mapping because it was there that it was derived; note also that on the vertical meridian the log mapping does not work well and needs to be extended by a shearing function there for preserving area constancy across meridians; Schira et al., 2007, 2010). Whitney and Levi (2011) include the cortical-constancy claim in their discussion of Bouma’s rule. Nandy and Tjan (2012, p. 465 and Online Methods) took the log mapping approach one step further and derived that the cortical equivalent (“the footprint”) of critical distance amounts to about six hypercolumns. The answer to the question what Bouma’s law looks like in the cortical map is of interest for our understanding of cortical architecture but is also of practical use for research; Mareschal et al. (2010), for example, applied the constancy assumption to their question and analysis of contextual influences on perceived orientation. Beware that a different, but slightly erroneous, nonconstant cortical critical distance rule was derived in Strasburger et al. (2011, Equation 28) and Strasburger and Malania (2013, Equation 13).

The constant-cortical-distance rule is appealing for its elegance and simplicity, and (for the horizontal meridian) its derivation in Pelli (2008) is mathematically sound. It needs, however, to be qualified—the constancy does not hold for the fovea, and that is sometimes overlooked. Looking closer, Schwartz (1980) has presented two logarithmic mapping functions, a general, and a simplified version. The latter is undefined in the centre (it omits a constant term in the log’s argument) and was meant to be applied only for eccentricities sufficiently above zero. It is the latter version, together with the simplified Bouma law (Figure 4A), that Pelli (2008) used in his derivations (and Pelli warns against this limitation).

A corrected rule for the horizontal meridian that includes the fovea is presented in Strasburger (2017c, 2019), shown in Figure 17. It was derived from the *cortical location function* which maps retinal location to cortical location and, as shown in that article, can be stated as

(7) $$d=\frac{d_{2}}{\ln2}\ln\left(1+\frac{E}{E_{2}}\right)$$

The dependent variable *d* in that equation is the distance on the cortical map from the retinotopic centre (*d*_0_), in millimetres, and the equation expresses it as a function of eccentricity *E* in the visual field, in degrees visual angle. There are two parameters in the equation, *E*_2_ and *d*_2_. The first, *E*_2_, is Levi’s value specifying at which eccentricity in the visual field the foveal value (of, e.g., MAR) is doubled (Levi et al., 1984; Strasburger et al., 2011; see Notes 5 and 8). The newly proposed parameter *d*_2_ is *E*_2_’s counterpart in the cortical map: the distance of the *representation* of *E*_2_ in the map from the retinotopic centre (that centre is roughly located at the occipital pole). *d*_2_ is a single empirical parameter, with a natural interpretation, that links the 1D cortical scale to the visual scale. From the location function (Equation 7), one can derive critical distance on the cortical map. One simply inserts the locations for target and flanker at the critical distance, for some target eccentricity *E,* and takes the difference. After simplification one obtains

(8) $$\kappa =M_{0}{\overset{E}{\;}}_{2}\ln\left(1+\frac{\delta_{0}}{{\overset{E}{\;}}_{2}}\frac{\left(1+\frac{E}{{\hat{E}}_{2}}\right)}{\left(1+\frac{E}{{\overset{E}{\;}}_{2}}\right)}\right)$$

Critical distance on the cortical map is denoted by kappa (κ) in the equation. Further parameters are *M*_0_: the cortical-magnification factor at the retinotopic centre (about 30 mm/deg), *δ*_0_: the centre-to-centre critical distance for crowding in the fovea centre (in deg visual angle), and a new parameter, *Ê_2_*: the *E*_2_ value for critical distance in Bouma’s law. About the latter: As said earlier (in the text after Equation 2), Bouma’s law is a linear function and is formally equivalent to *M* scaling. It can thus be written in the standard *E*_2_-notation as

(9) $$\delta =\delta_{0}\left(E/{\hat{E}}_{2}+1\right)$$

The *Ê_2_* in that equation is the eccentricity in the visual field at which the critical-distance value in the centre (*δ*_0_) doubles (or, equivalently, is the eccentricity increment at which critical distance increases by the foveal value, *δ*_0_).

The graph of Equation 8 is shown in Figure 17B. Critical distance for crowding on the cortical map starts at some value in the retinotopic centre (i.e., at *E* = 0°), and then—depending on the ratio *E*_2_/*Ê*_2_ (the ratio of the respective *E*_2_ values for MAR and crowding)—quickly increases to a different value that it reaches asymptotically. Constancy is thus reached above some eccentricity value, probably somewhere just outside the fovea. This equation can thus be seen as a generalization of Pelli’s result, which now also covers the case of central vision and reading.

### Summary 6

The assumption of an essentially constant cortical distance is not yet frequent in the literature, and authors are aware that it is a simplification that is not valid in the fovea. Still, it should be helpful to know that an empirically valid rule including the fovea can be derived from first principles.

## Crowding Research

*Misconception 7*: Except for Bouma’s (1970) seminal article, crowding research mostly became prominent starting in the 2000s.

Crowding is “quite the rage” in vision research these days; a very modern enterprise it is. The previous statement is of course a caricature, but I do feel that the strong pertinent research tradition from the 60s, 70s, and 80s, as well as the initial article by Korte (1923), do not get the credit they deserve. Not only are articles from that time rarely cited, many scholars also do not know what is said there (and are blissfully unaware that what is reported in them might precede one’s own ideas—after all, it is good scientific practice to give the credit to whoever said it first).

A simple reason for that neglect might have been that other terms for the phenomenon, or similar or related phenomena, were the popular ones at those times and consequently do not show up in a search for *crowding* as a keyword: *Lateral masking*, *lateral inhibition*, *lateral interference*, *interaction effects*, *contour interaction*, *surround suppression*, *mutual or cognitive inhibition* (Strasburger et al., 1991; Danilova & Bondarko, 2007).

Obviously, the terms in that list denote somewhat different concepts and phenomena, and, indeed, there are important differences between them and to what we might call prototypical crowding. A number of authors have in the past worked out criteria to disentangle the phenomena (e.g., Levi et al., 2002b; Pelli et al., 2004; Huckauf & Heller, 2004; Petrov et al., 2007; Lev & Polat, 2015). Yet even though certain distinctions appear fairly reliable (e.g., detection vs. recognition of the target in a flanked task, dependence of performance on, vs. independence of, target size), the usage of the terms in that list is not consistent enough to justify an exclusion of any of these in a literature review. And, in particular with respect to the older literature, the meaning of the terms has slightly changed over time. That is not to say such attempts of clarifying the concepts were fruitless or not important, quite to the contrary. It just means that we still lack a coherent theory of crowding that determines what *is* crowding, and what is *not*. In any case, one is surprised what shows up with these keywords in standard search machines.

Another, somewhat trivial reason for the neglect, at least for a while, might have been that full-text versions of older articles were not available online. I still have my collection of reprints from the 1980s and 1990s. In the comparably young history of crowding research, that change of reading and writing habits away from printed material must have had an influence. Digitization of the older literature is not complete (e.g., *Clinical Vision Sciences* is missing); that of the 19th century and before is still an ongoing process (a good source for the latter is the *Internet Archive*, https://archive.org/, from where we retrieved historic articles by Helmholtz, Volkmann, and Wülfing, for Strasburger, Huber, & Rose, 2018).

**Figure 18.**
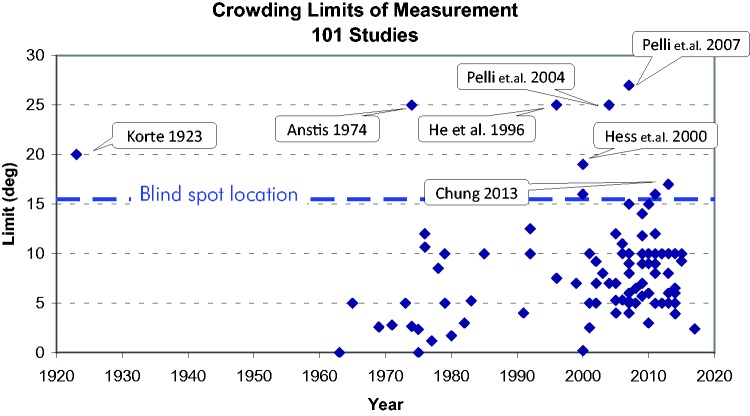
Essential Crowding Literature From 1923 to 2004. Abscissa: year of publication; ordinate: eccentricity in the visual field up to which crowding was studied in the article. (References given in Figure 19 up to 2004.)

Figure 18 shows a chart of crowding literature up to the present. Note it is by no means complete. The *x* axis shows the year of publication and the *y* axis the maximum eccentricity (on a meridian or in the visual field) up to which data were reported. The horizontal dashed line at 15.5° marks the blind spot (on the horizontal meridian) as a reference (Rohrschneider, 2004).

There are four points I wish to make: (a) The vast majority of studies are concerned with quite small eccentricities (cf. Misconception 2). (b) The maximum eccentricity up to which crowding was studied is a mere 25°. Given that pattern recognition is possible in most all of the visual field and has been proven to be so up to about 80° for simple forms (Collier, 1931; Menzer & Thurmond, 1970; Strasburger, 2017a), one wonders what crowding is like beyond 25°. (c) With respect to the year 2000: Indeed, research “took off” at around 2000, but there are quite a number of publications in the 70s to 90s. (d) The time span between 1923 and 1962 is curiously empty in the graph (Ehlers, 1936, 1953, are not listed as they present no data). Filling the gap might need more digging in the older literature. Another reason from that break, however, could be the expulsion of Gestalt psychologists from Germany, who were those interested in visual phenomena at that time.

**Figure 19.**
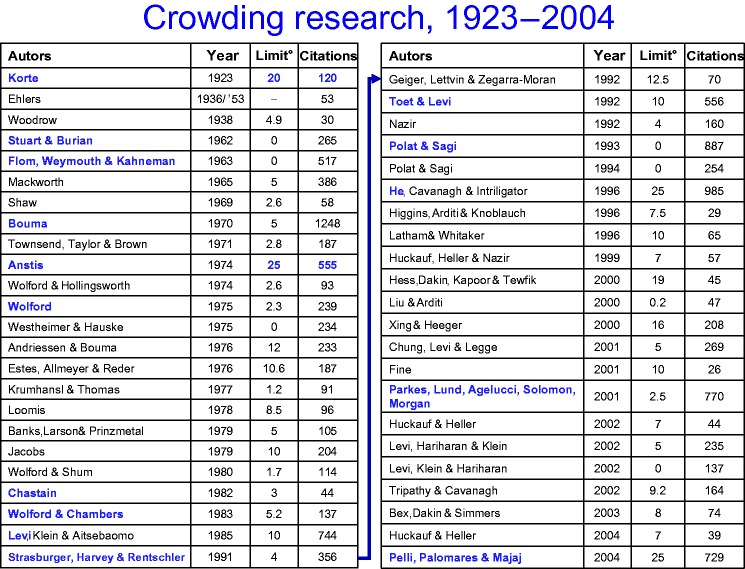
Crowding Literature From 1923 to 2004 Shown in Figure 18. Bold blue print: particularly important articles (in my subjective assessment). The column *Limit °* shows (as before) the eccentricity of the target in the visual field up to which crowding was studied. The last column shows the number of citations from a Google Scholar search (November 2019).

Figure 19 gives the references for the articles in that graph up to 2004. Those in bold print might be seen as landmark articles, but this is of course a subjective view (and is not always borne out by the number of citations, given in the last column).

### Crowding Research Before 1923

Ehlers (1936) in the previous list is the first documented use of the term *crowding*; the Gestalt psychologist Wilhelm Korte (1923) was the first who provided an analysis of phenomena in indirect vision including phenomena related to crowding (see Strasburger, 2014 for an excerpt). What happened on crowding before that?

**Figure 20.**
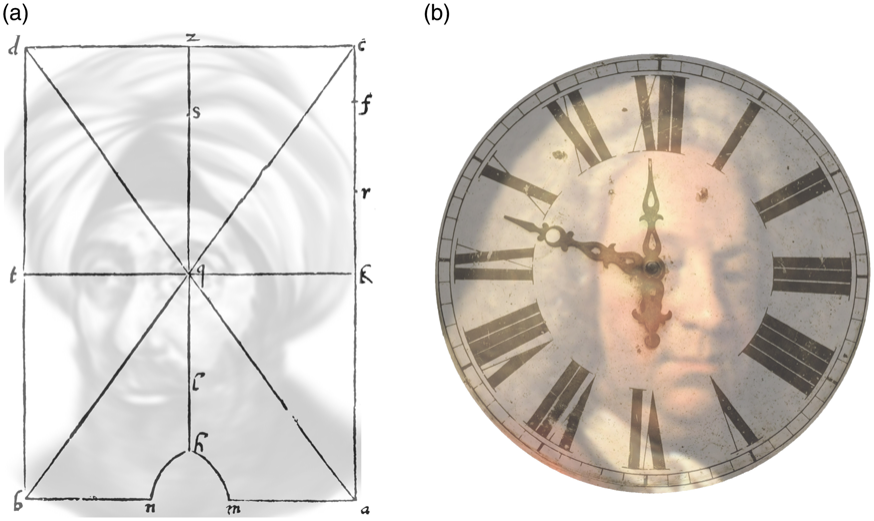
(A) Portrait of Ibn al-Haytham (c. 965 to c. 1040), with his perimeter superimposed (from Strasburger & Wade, 2015b). (B) Portrait of James Jurin (1684–1750) with a clock face superimposed, as the one described in his text and common at the time. Note that the number *Four* is not in correct roman notation, so crowding will have been more prominent (from Strasburger & Wade, 2015a; both artworks by Nicholas Wade, 2015).

Surprisingly, phenomena that today we would interpret as *crowding* were already described in writing a thousand years ago, by Ibn al-Haytham (latinized *Alhazen*; 965–1039, Figure 20A, Strasburger & Wade, 2015a). This is as early as vision was explained, like today, “as the outcome of the formation of an image in the eye due to light” (Russell, 1996, p. 686; before that, vision was explained by rays emanating *from* the eye).

Here is a description from al-Haytham’s “Optics”:

The experimenter should then gently move the strip [**with a word written on it**] along the transverse line in the board, making sure that its orientation remains the same, and, as he does this, direct his gaze at the middle strip while closely contemplating the two strips. He will find that as the moving strip gets farther from the middle, the word that is on it becomes less and less clear…and decreases in clarity until [the observer] ceases to comprehend or ascertain its form. Then if he moves it further, he will find that the form of that word becomes **more confused and obscure**. (Ibn al-Haytham, translated in Sabra, 1989, pp. 244–245, cited after Wade, 1998; emphasis added)

Importantly, al-Haytham used words, not single letters, in that experiment. So the “confused and obscure” percept that he describes arises from crowding. The only ingredient missing for an experimental unveiling of the crowding phenomenon was a direct comparison with single letters at the respective eccentric location, which he could have easily done with his apparatus.

A second example for a close miss is James Jurin’s *An Essay on Distinct and Indistinct Vision* (1738; Strasburger & Wade, 2015a, Figure 20B). For explaining visibility, Jurin observes, “173. […] The more compounded any object is, or the more parts it consists of, it will, ceteris paribus, be more difficult for the eye to perceive and distinguish its several parts” (Jurin, 1738, p. 150).

This would appear an apt characterization of the crowding phenomenon, in particular when the text continues as,

175. From the same cause of the instability of the eye it must be, *ceteris paribus,* more difficult to perceive and distinguish the parts of any compound object, when each of those parts subtends a very small angle, than to see a single object of the same magnitude as one of those parts. (p. 151)

However, the examples that follow in Jurin’s essay, even though related to crowding, would not be considered typical for crowding today:

173. […] For instance, it is somewhat difficult for the eye to judge how many figures are contained in the following numbers, 1111111111; 1000000000. But if we divide the figures in this manner, 11111,11111; 10000,00000; so as to constitute several objects less compounded, we can more easily estimate the number of figures contained in each of those numbers; and more easily still, if we thus divide them, 1,111,111,111; 1,000,000,000. (Jurin, 1738, p. 150)

A rough estimate shows that, at normal reading distance (30 cm), these patterns have around 4.5° extent and 0.5° centre-to-centre letter distance and are thus expected to undergo crowding. Jurin’s observation that segmentation helps in the recognition reminds us of the end-of the-word effect explained earlier and the importance of separators. Yet unlike in crowding, all the numerals in the strings are unambiguous, and the difficulty is rather one of perceiving their correct number. Yildirim et al. (2020) have called that phenomenon *redundancy masking*, which they argue is related to, but not the same as crowding. Note also the use of separators (Shaw, 1969; Estes & Wolford, 1971).

A second example in the treatise refers to a clock face: “175. […] For instance, the hour I. upon a dial plate may be seen at such a distance, as the hours II, III, IIII, are not to be distinguished at, especially if the observer be in motion” (Jurin, 1738, p. 151).

From the end of the latter quote (and what follows in the essay), Jurin is at a loss of explaining the phenomenon by ray tracing (as he does in all other of his many examples) and instead invokes self-motion for an explanation. Thus, even though Jurin comes close to discovering the phenomenon—by virtue of his very careful description of visual phenomena and his concept of *indistinct vision*—he finally stays with the contemporary way of analysis based on a blurred retinal image (cf. Strasburger, Bach, et al., 2018).

### Summary 7

The study of crowding in today’s sense started, from what I can see, with Stuart and Burian’s (1962) *A study of separation difficulty* on amblyopic vision. The phenomenon has been known much earlier to ophthalmologists and optometrists, as is apparent from Ehlers’s (1936, 1953) comments, yet I am not aware of an earlier treatise from those fields. Korte’s (1923) *Über die Gestaltauffassung im indirekten Sehen* was the first to describe the phenomena of form perception for letters and words in near-peripheral vision, including what we now call crowding. Korte, after he obtained his degree in Leipzig in 1922, apparently did not pursue a further scientific career. His treatise is not translated, but a summary can be found in Strasburger (2014). The 1960s to 1990s were a busy time for crowding research, mostly from experimental psychology. However, in that time—with a few exceptions—the term *crowding* was not used. So that sometimes gives the impression that nothing much happened then, and the field only really took off after the turn of the century.

Curiously, even though the crowding phenomenon can be easily demonstrated on a paper napkin, without any apparatus, it apparently was not described earlier than Korte (1923). Alhazen in the 11th century came close, when he describes how a written word in peripheral vision becomes *confused and obscure*. We will readily agree with this today.

## Conclusion

So should we care? Much of what was said previously might be obvious. Or, on the other end of the spectrum, one might disagree with some points. The points made earlier are also not all equally important and are not all of general interest. However, once a myth has found its way into a textbook, it is very hard to remove it for good (cf. Wilkes, 1997; Wade & Tatler, 2009). Not only that, it will also spread—like a virus, unfortunately. Textbook authors copy from other textbooks. Scientific authors copy from textbooks. Wikipedia excerpts from textbooks. Lecturers take their materials mostly from textbooks. We probably all know examples.^30^ Thus, vision scientists better discuss the obvious in time and weed out the shady parts and the fluff. I thus wish to invite my readers to a discussion and hope for many more articles on myths.

## Supplemental Material

sj-pdf-1-ipe-10.1177_2041669520913052 - Supplemental material for Seven Myths on Crowding and Peripheral Vision Click here for additional data file. Supplemental material, sj-pdf-1-ipe-10.1177_2041669520913052 for Seven Myths on Crowding and Peripheral Vision by Hans Strasburger in i-Perception

## Notes

1 Talk slides for this article were published as preprint in Strasburger (2018).

2 “It has been stated in the past (Ehlers, 1936, 1953; Adler, 1959) that the crowding phenomenon occurs in normally sighted people. However, no quantitative data have thus far been available, except for the study of Thomas-Decortis (1959) which will be discussed later.” (p. 471)

3 “When one is testing amblyopic children with isolated letters or E’s, the visual acuity recorded is often much better than with the ordinary test chart. If the visual field is crowded with letters, the area of the visual field in which the letters can be recognized narrows. This is very easy to demonstrate, as I showed at the Congress of Scandinavian Ophthalmologists in 1936.” (Ehlers, 1953, p. 432)

4 *Indirect vision* is a term describing vision off the point of fixation. It is often used synonymously with *peripheral vision* but has a different emphasis (i.e., seeing off-centre). See the appendix in Strasburger (2014) for a discussion of these terms.

5 The *E*_2_ value (Levi et al., 1985) is a measure for normalized slope of a visual capacity’s dependence on eccentricity. It is defined as the eccentricity where its foveal value doubles or, equivalently, as the eccentricity increment for which the visual parameter in question increases by the foveal value (see Strasburger et al., 2011, Section 3.2, for review).

6 Personal communication at the 18th European Conference on Visual Perception, Tübingen, 1995.

7 As explained later, *foveal vision* and *vision in the fovea* mean something different. The fovea is about 5° in diameter; the debate about whether *foveal crowding* exists, in contrast, refers to the central 0.1°.

8 The caption says “An eye chart in which letters in different parts of our visual field have been scaled to make them equally legible. The size has to double approximately every 2.5° in order to do this.” This innocent sounding description is formidably incorrect in two ways: (a) The 2.5° value is meant to be the *E*_2_ value (Levi et al., 1985), but its definition is misunderstood. It is defined as a doubling *of the foveal* value, not a doubling *every* 2.5° as said in the caption. The doubling rule would lead to an exponential increase (*y* = 2^n^ · s_0_ with *n* being the number of increments), not to a linear function as required. Interestingly, that misconception might be more widespread; the same mistaken use of “every” is found in Latham and Whitaker (1996, p. 56), so perhaps it would warrant a myth of its own. (b) The graph is modelled after Anstis’s exaggerated version (see Misconception 3), so it is scaled 10-fold as steep as required for legibility. Note also that that image is already crowded, as explained later.

9 For example, Herzog, M., and Sayim, B. (2019, June 23–24). Workshop on visual crowding, Murten, Switzerland.

10 To quote from this educational essay, “Student knowledge, however, can be erroneous, illogical or misinformed. These erroneous understandings are termed **alternative conceptions or misconceptions** (or intuitive theories). Alternative conceptions (misconceptions) are not unusual. In fact, they are a normal part of the learning process.”

11 On the more general subject of myths in neuroscience and what they have to do with occult passions, one will enjoy *The frog’s dancing master* by Piccolino and Wade (2013). Or about the myth that the high iron content in spinach originated from a misplaced comma, you will be surprised to learn that that itself is a myth (Rekdal, 2014).

12 “For a stimulus at φ° eccentricity, an open distance of roughly 0.5 φ° is required for complete isolation” (Bouma, 1970, p. 177).

13 The two numbers refer, respectively, to the critical flanker distance below which a transient ring cue around the target does not improve its contrast threshold, and the point of maximum cue gain control effect, described by Equations 5 and 6 in that article.

14 The shearing-function model of the cortical map by Schira et al. (2007, 2010) would predict deviations from linearity on the vertical meridian at around 1° eccentricity (cf. Schira et al., 2010, Figure 2). These might have been missed because one did not specifically look for them.

15 For the importance of empty space for recognition, see Shaw (1969) and Estes and Wolford (1971).

16 Pelli et al. (2007) use this equation with the foveal value of *d* for the constant term *w*, saying it is “about 0.1° or 0.2°” (p. 6). They call the slope constant *Bouma’s factor*.

17 The somewhat overly precise figure of 5.2° given in Polyak’s book and Wandell’s (1995) summary stems from converting a rounded diameter of 1,500 micrometers on the retina to degrees visual angle.

18 H. Wässle, personal communication, 7 August 2019; there is no precise border, so estimates vary widely.

19 For conversion: 3.43 deg/mm (cf. Le Grand, 1957, p. 50).

20 Coates et al. (2018) also isolate a separate recovery mechanism, first observed by Flom et al. (1963), at even smaller distances – 0.5–0.75 arcminutes. We can leave that aside for the present discussion.

21 The kink in the hockey stick is at *s* = 5’. The bar is at 4’ eccentricity from the graph; it has the same stroke width as the letter, *s*/5 = 1’. The gap (empty space) *g* thus extends from 2.5’ to (4’ minus 0.5’), that is, is 1’ wide.

22 As to the clinical relevance, the so-called *temporal crescent* (starting at an eccentricity of approx. 50° and extending to more than 90°) is indeed of neuro-ophthalmological importance, contrary to a widespread assumption: Losses in that area indicate the affection of postchiasmal fibers, emanating from the contralateral peripheral nasal retina. Typical locations for lesions are the contralateral Meyer’s loop or the contralateral deep-rostral portion of the striate cortex (U. Schiefer, personal communication, 22 July 2019).

23 The negative constant of –0.031 in Anstis’s equation stems from linear regression and is physically meaningless. It could be replaced by the foveal MAR value which, because it is small, would leave the slope of 0.046 unchanged. Note that if we would set the constant to 0 and use *S* = 0.046 *E* for simplicity, that equation could no longer be converted to an *E*_2_ scheme. Neither could it be described by *M* scaling like in Equation 2. The reason is the difference between proportionality and a linear law, explained earlier (under Misconception 1).

24 It should not be confused with a *temporal-nasal* asymmetry, as is sometimes the case, because it refers to the visual field, not the retina.

25 Here are a few examples how the asymmetry is phrased: “The adverse interaction is stronger if the interfering /x/ is at the peripheral side” (Bouma, 1970, p. 178). “A similar asymmetry [of reportability] appeared on the central-peripheral dimension in the visual field. […] [Many more] reports were correct on letters immediately central to a space” (because spaces might “function … as attenuators of lateral masking effects of neighboring characters”; Estes & Wolford, 1971, pp. 77, 78, 79). “A more peripheral flanking element crowded more effectively than a more foveal one” (Bex et al., 2003, p. 2895). “Crowding is directed to the fovea”; “the outward element was crowded much less than the inward elements” (Petrov & Popple, 2007, p. 5). “There is a further ‘centrifugal anisotropy’ such that flankers that are nearer to fixation can get closer to the target without interfering with identification than more eccentric flankers” (Dakin et al., 2010, p. 2). “More peripheral distractors exert a greater impact on more foveal targets than vice-versa” (Dayan & Solomon, 2010, p. 2254). “It has long been known that an outward mask is much more disruptive than an inward mask in crowding (H. Bouma, 1973)” (Petrov & Meleshkevich, 2011b, p. 1).

26 “The beginning and ending letters of the word are perceived correctly even when nothing else can be reported correctly” (Haslerud & Clark, 1957, p. 99).

27 Note that Toet and Levi (1992) used flankers on either side of the target, whereas Bouma’s (1970) pilot data were based on using only one flanker. Note further that the asymmetry implies that those elongated fields are asymmetric along the radius.

28 Note that the feature concept in Dakin et al. (2010) is different from the one used here or that in Wolford’s (1975) feature-perturbation model.

29 A (symmetric) model of word recognition that very successfully treats location errors and identification errors separately was recently presented by Bernard and Castet (2019). To quote from the article, “This result suggests that letter position uncertainty is an important and overlooked factor limiting peripheral word recognition (and reading without central vision in general)” (p. 57).

30 Is there a Weber-Fechner law? Or is that a *textbook hoax*? (in German, a “*textbook duck*”). Or, which term is correct: “chi-squared” or “chi-square”?
