# Supplementary material for: Seven Myths on Crowding and Peripheral Vision
Source: Iperception. 2020 May 19;11(3):2041669520913052. doi: 10.1177/2041669520913052 (PMC7238452; doi:10.1177/2041669520913052)
Supplement: sj-pdf-1-ipe-10.1177_2041669520913052 - Supplemental material for Seven Myths on Crowding and Peripheral Vision [file sj-pdf-1-ipe-10.1177_2041669520913052.pdf]

Answers to Reviewers, Revision 2

Reviewer: 2

Prof. Strasburger has again undertaken a considerable series of revisions to this review, and the issues/misunderstandings that I had raised earlier are now resolved. The review is comprehensive and wide-reaching, with a range of useful reference points for researchers in the field. There are a few minor typographic errors, noted below, but I have no further comments at a substantive level.

Thanks.

Page/line numbers refer to the document with tracked changes:

-p3 line 52 'here' should be 'where'. Done

-p4 line 41 'those' should be 'these'. Done

-p7 line 41 'todisprove' needs a space. Done

-p22 line 6 should be either '…peripheral vision degrades faster because of crowding' or '…peripheral vision degrades because of greater crowding'.

A quotation looked strange because it was incomplete. I now give the full sentence, with page number.

-p31 line 45 'that kind' should be 'the kind'. Done

-p31 line 46 'with now' should be 'now with'. Done

-p34 line 31 'literature' is misspelt. Done

-p34 line 52 should be 'as a keyword'. Done

-p38 line 38 'Yildirim' is misspelt. Done

-p38 line 54 should be 'crowding in today's sense'. Done

Reviewer: 1

The changes in the revised version significantly improved the manuscript. I only have a few suggestions and some minor points.

The number of footnotes is rather high. A reduction both in number and length would improve readability. Some of the footnotes could equally well be mentioned in the main text (if they are considered necessary in the first place). For example: "In peripheral vision [at an eccentricity of 9.2° in the lower visual field], a 5-fold change in target size produced less that a 15% change in the spatial extent of interaction". (Tripathy & Cavanagh, 2002, Fig. 4 and p. 2365)".

Footnotes were revised and their number reduced a little (it's not visible in the pdf since Word does not handle footnote numbering in track-changes mode).

Some statements should be clarified.

** Page 3, line 22: The negation "not always" following "doing away with" could be misleading: "It does away with centuries of two core assumptions in visual perception (cf. Strasburger & Wade, 2015a), namely that good vision comes down to good acuity, and, more generally, that a reductionist approach is not always (or necessarily) the best way for solving a scientific problem".*

Thanks. Rephrased.

** Page 5, line 8: What does it mean if the selection is entirely (!) subjective? "And finally, my selection here is entirely subjective; the points are a way of summarizing my unease while reviewing papers on crowding or trying to put things into perspective".*

Changed to: "Their selection reflects what I found interesting and noteworthy."

** Page 35, line 6: What "terms" are meant exactly: "Yet even though certain distinctions appear fairly reliable (e.g. detection vs. recognition, dependence on, vs. independence of, target size), usage of the terms is not consistent enough to justify an exclusion of any of these terms in a literature review". Unclear: "It just means that we still lack a coherent theory of crowding".*

It refers to the list of terms before that paragraph. The paragraph is rephrased. One term is added (from Danilova & Bondarko's 2007 list).

** Page 15, line 12: Makes it sound as if the physical stimulus size is simply measured, but there is observer data to determine the MAR: "Put that way, there is no answer to that question because the MAR and the crowding effect are measured on different dimensions (stimulus size vs. proportion correct)".*

That statement was in the previous revision (R1) and was rephrased in R2. Anyway, it is rephrased again to prevent that possible misunderstanding.

A few speculative statements should be rephrased:

** Page 2, line 47: "Oddly, vision research – which, as the highly interdisciplinary field that it is, could have been the unifying ground – with a few exceptions appeared not interested. Neither were the cognitive sciences or visual neuroscience".*

Rephrased.

The section on binding (page 30) is rather speculative and a manuscript on myths in crowding might not be the right place for it.

The explanations became necessary to prevent misunderstandings in the context of crowding asymmetry and because (letter) features must not be confused with (letter) parts.

A few remarks on references:

There is a large literature on foveal crowding, and the strong focus on the hockey stick model seems to come with a cost for other relevant work. A few examples:

Badcock and Westheimer, 1985; Danilova and Bondarko, 2007; Ehrt and Hess, 2005; Hess, Dakin and Kapoor, 2000; Jacobs, 1979; Lev, Yehezkel and Polat, 2014; Loomis, 1978; Nazir, 1992; Polat and Sagi (1993, 1994); Sayim, Westheimer and Herzog (2008, 2010)

Thanks for the suggestions. To keep the focus on the main point, the references were simply added in the introductory list of work.

Badcock and Westheimer, 1985: There are two papers with that year (Vis. Res. and PNAS), both a little peripheral to what is discussed here.

Danilova and Bondarko, 2007: Nice review, and suggests a term I was not aware of. Included.

Ehrt and Hess, 2005: Difference between detection and discrimination. Included

Hess, Dakin and Kapoor, 2000: important but disproved by Danilova & Bondarko (2007). Hess, Dakin, Kapoor & Tewfik (2000) is similar and cited.

Jacobs, 1979: Early comparison of fovea and periphery. Included.

Lev, Yehezkel and Polat, 2014: much larger target-flanker spacing with short presentation times. Included.
Loomis, 1978: Flom task in the fovea and periphery and a review of the old work. Included.
Nazir, 1992: Target-mask similarity. Included.
Polat and Sagi (1993, 1994): 1993: suppressive vs. facilitatory region. Included. 1994: There are two in 1994, Vis. Res. and PNAS. I chose the latter, which shows the influence of learning.
Sayim, Westheimer and Herzog (2008, 2010): Further references to the Gestalt story are cited in M1.

Examples from more recent and more widely used text books could be selected. For example, the German version of Goldstein (2002) seems not to be ideal.

Excellent suggestion. I realized that I was not aware of two newer textbooks on the American market and found that Wolfe et al. (2019) indeed covers crowding. I added a paragraph on the selection of books. The graph from the German Goldstein version was replaced by the figure from the American version and the text changed accordingly.

When discussing Bouma's law versus Bouma's rule of thumb, previous contributions discussing the main topic should be mentioned, for example, Whitney and Levi, 2011.

Thanks for the suggestion; I was not aware of that section in Whitney and Levi (2011). The paper is now mentioned in that discussion and further in the section on the constant cortical distance for crowding.

References in Figure 18 are missing in Figure 19: Chung, 2013; Pelli et al., 2007

Figure 19 only goes to 2004, as said in the caption. That restriction is now also mentioned in the main text. The newly added papers from the revision (if before 2004) are now also added in Figure 19.

Some seemingly contradictory statements:

** Page 14, line 6: "It is present, and in a sense even more important, in the foveal area of around five degree diameter".*

Added, "even though particularly pronounced in the periphery"

** Page 14, line 43: "Crowding is particularly pronounced in peripheral vision, so we should..."*

Solved by the above.

** Page 21, line 52: "Crowding is thus generally much more important as a limit to pattern recognition, even in the foveal area".*

Changed, "even in the foveal area" to "even already in the foveal area".

** Page 25, line 33: "Up to some small eccentricity, there is no crowding at all in most scenes".*

Moved the qualifier "in most scenes" forward, and added an explanation:
"Up to some small eccentricity, in most scenes, there is no crowding at all (since adjacent contours are sufficiently far away)."

Colloquial:

** "I still have my collection of reprints from the 1980s and 90s".*

Changed to: "Not too long ago, researchers relied on libraries and author reprints for literature reviews."

** Page 10, line 40: “Isn’t that beautiful?”*

Well, it is meant to be colloquial in that context and I would like to keep it.

The section necessary on crowding research before 1923 is rather long and could be shortened or deleted.

I fear it would not be clear if shortened, and think that two manuscript pages including images and summary for a thousand years review should be acceptable (I deleted a few words).

A few minor points:

** Page 2, line 17: How does the demonstration show that it is a “general phenomenon of visual perception"?*

Rephrased.

** Figure 1: The left duck has a signature, delete or include for the other ducks?*

It is an original piece of art by a known artist, commissioned for this paper. Therefore, I would like to keep the signature in the original image on the left.

** Figure 5: Why “degenerated”, it's simply overlapping.*

Yes. But with overlapping, it’s no longer a crowding task. Phrasing slightly changed.

** The references to the figures by Anstis are complicated, for example: “Anstis’s third chart (Fig. 5, shown in Figure 9c)...”*

Agreed. Except for the opening sentence on Anstis’s figures, the original figure numbers are removed for clarity.

** A few figures are missing the numbering mentioned in the captions (for example 9a, b and c).*

Labels (a, b, c) are now added to all figures where there is more than one figure part.

** Page 7, line 27: “additional flankers surrounding the standard task have little or no influence”*

Changed to, “additional flankers on the left and right have little or no influence”.

** Figure 10: Explicitly mentioning what is the scale for the MAR in the caption would be helpful.*

Well taken. A sentence is added to the caption.

** Page 36, line 21: It's not bold but blue print.*

It is both. Changed to “bold blue print”.
